# Supplementary material for: 1.25(OH)2D3 decreases PCNA and mTOR expression and alleviates renal injury in Thy-1 nephritis rat model
Source: PLoS One. 2024 Dec 5;19(12):e0311000. doi: 10.1371/journal.pone.0311000 (PMC11620346; doi:10.1371/journal.pone.0311000)
Supplement: Attachment — Submitted filename: PONE-D-24-22668_R1.pdf [file pone.0311000.s002.pdf]

PLOS ONE

1.25(OH)2D3 decrease PCNA and mTOR expression and alleviates renal injury in Thy-1 nephritis rat model

--Manuscript Draft--

**Manuscript Number:**	PONE-D-24-22668R1
**Article Type:**	Research Article
**Full Title:**	1.25(OH)2D3 decrease PCNA and mTOR expression and alleviates renal injury in Thy-1 nephritis rat model
**Short Title:**	1.25(OH)2D3 regulates the proliferation of glomerular mesangial cells
**Corresponding Author:**	Xiao-ping Yang<br>First Affiliated Hospital of Shihezi University School of Medicine<br>Detroit, CHINA
**Keywords:**	1.25(OH)2D3; mammalian target of rapamycin (mTOR); Thy-1 Nephritis; Proliferation
**Abstract:**	Objective: This study explored the role and mechanisms of 1.25(OH)2D3 in proliferative glomerulonephritis and its effect on the regulation of mesangial cells.<br>Methods: Sixty male SD rats were randomly divided into four groups: control (CG), nephritis (NG), nephritis + 1.25(OH)2D3 (NVG), and nephritis + 1.25(OH)2D3 + rapamycin (NVRG) (n=15 per group). Three rats from each group were sacrificed on days 1, 3, 7, 14, and 21 after intervention. Urine samples were collected from the rats over 24 hours on day 0 to measure urinary protein excretion. Renal tissue samples were stained with HE and PAS to assess the severity of renal injury, and immunohistochemistry was used to measure PCNA and mTOR expression in the renal tissues.<br>Results: Compared to NG, mesangial cell proliferation in the renal tissues was significantly alleviated in NVG and NVRG at all time points (all $p<0.05$); PCNA expression was significantly higher in the NG compared to the CG ($p < 0.05$) and significantly lower in the NVG and NVRG ($p < 0.05$). mTOR expression was also significantly increased in the NG compared to the CG, with a significant reduction observed in the NVG and NVRG compared to the NG.<br>Conclusion: Our findings clarify that 1.25(OH)2D3 could significantly inhibit the proliferation of glomerular mesangial cells in rats. Also, mTOR protein was involved in the process of 1.25(OH)2D3 regulating glomerular mesangial cells. Our resuits further elucidate the molecular mechanism of 1.25(OH)2D3 in alleviating renal injury in glomerulonephritis.
**Order of Authors:**	Jian-feng Li
	Lei Jin
	Huan Ma
	Jie Suo
	Rui Yang
	Xiao-ping Yang
**Opposed Reviewers:**	
**Response to Reviewers:**	Thank you for carefully reviewing our manuscript.<br>Weare grateful to all reviewers for their constructive critique.<br>Thank you for your recognition and encouragement.
**Additional Information:**	
**Question**	**Response**
**Financial Disclosure**<br><br>Enter a financial disclosure statement that describes the sources of funding for the	Yes

work included in this submission. Review the submission guidelines for detailed requirements. View published research articles from *PLOS ONE* for specific examples.<br><br>This statement is required for submission and **will appear in the published article** if the submission is accepted. Please make sure it is accurate.<br><br>**Funded studies**<br>Enter a statement with the following details:<br>• Initials of the authors who received each award<br>• Grant numbers awarded to each author<br>• The full name of each funder<br>• URL of each funder website<br>• Did the sponsors or funders play any role in the study design, data collection and analysis, decision to publish, or preparation of the manuscript?<br><br>Did you receive funding for this work?	
Please add funding details.<br>as follow-up to "**Financial Disclosure**<br><br>Enter a financial disclosure statement that describes the sources of funding for the work included in this submission. Review the submission guidelines for detailed requirements. View published research articles from *PLOS ONE* for specific examples.<br><br>This statement is required for submission and **will appear in the published article** if the submission is accepted. Please make sure it is accurate.	This work was supported by the National Natural Resources Foundation of China (81160090) and Research Project Supported by Xinjiang Production and Construction Corps of China (2009GG59)

Powered by Editorial Manager® and ProduXion Manager® from Aries Systems Corporation

**Funded studies**<br>Enter a statement with the following details:<br>• Initials of the authors who received each award<br>• Grant numbers awarded to each author<br>• The full name of each funder<br>• URL of each funder website<br>• Did the sponsors or funders play any role in the study design, data collection and analysis, decision to publish, or preparation of the manuscript?<br><br>Did you receive funding for this work?"	
Please select the country of your main research funder (please select carefully as in some cases this is used in fee calculation).<br>as follow-up to "**Financial Disclosure**<br><br>Enter a financial disclosure statement that describes the sources of funding for the work included in this submission. Review the submission guidelines for detailed requirements. View published research articles from *PLOS ONE* for specific examples.<br><br>This statement is required for submission and **will appear in the published article** if the submission is accepted. Please make sure it is accurate.<br><br>**Funded studies**<br>Enter a statement with the following details:<br>• Initials of the authors who received each award<br>• Grant numbers awarded to each author<br>• The full name of each funder<br>• URL of each funder website<br>• Did the sponsors or funders play any role in the study design, data collection and analysis, decision to publish, or preparation of the manuscript?<br><br>Did you receive funding for this work?"	CHINA - CN
**Competing Interests**	NO authors have competing interests

Use the instructions below to enter a competing interest statement for this submission. On behalf of all authors, disclose any competing interests that could be perceived to bias this work—acknowledging all financial support and any other relevant financial or non-financial competing interests.<br><br>This statement is required for submission and **will appear in the published article** if the submission is accepted. Please make sure it is accurate and that any funding sources listed in your Funding Information later in the submission form are also declared in your Financial Disclosure statement.<br><br>View published research articles from *PLOS ONE* for specific examples.<br><br>**NO authors have competing interests**<br><br>Enter: *The authors have declared that no competing interests exist.*<br><br>**Authors with competing interests**<br><br>Enter competing interest details beginning with this statement:<br><br>*I have read the journal's policy and the authors of this manuscript have the following competing interests: [insert competing interests here]*<br><br>* typeset	
**Ethics Statement**<br><br>Enter an ethics statement for this submission. This statement is required if the study involved:<br><br>• Human participants<br>• Human specimens or tissue<br>• Vertebrate animals or cephalopods<br>• Vertebrate embryos or tissues	All animal studies (including the mice euthanasia procedure) were approved by Shihezi University institutional animal care and done in compliance with the regulations and animal welfare guidelines of Shihezi University institutional animal care (SCXK (Xin) 2013-178).

Powered by Editorial Manager® and ProduXion Manager® from Aries Systems Corporation

• Field research<br><br>Write "N/A" if the submission does not require an ethics statement.<br><br>General guidance is provided below. Consult the submission guidelines for detailed instructions. **Make sure that all information entered here is included in the Methods section of the manuscript.**	

Powered by Editorial Manager® and ProduXion Manager® from Aries Systems Corporation

**Format for specific study types**<br><br>**Human Subject Research (involving human participants and/or tissue)**<br>• Give the name of the institutional review board or ethics committee that approved the study<br>• Include the approval number and/or a statement indicating approval of this research<br>• Indicate the form of consent obtained (written/oral) or the reason that consent was not obtained (e.g. the data were analyzed anonymously)<br><br>**Animal Research (involving vertebrate animals, embryos or tissues)**<br>• Provide the name of the Institutional Animal Care and Use Committee (IACUC) or other relevant ethics board that reviewed the study protocol, and indicate whether they approved this research or granted a formal waiver of ethical approval<br>• Include an approval number if one was obtained<br>• If the study involved *non-human primates*, add *additional details* about animal welfare and steps taken to ameliorate suffering<br>• If anesthesia, euthanasia, or any kind of animal sacrifice is part of the study, include briefly which substances and/or methods were applied<br><br>**Field Research**<br><br>Include the following details if this study involves the collection of plant, animal, or other materials from a natural setting:<br>• Field permit number<br>• Name of the institution or relevant body that granted permission	
**Data Availability**<br><br>Authors are required to make all data underlying the findings described fully available, without restriction, and from the time of publication. PLOS allows rare exceptions to address legal and ethical concerns. See the PLOS Data Policy and FAQ for detailed information.	Yes - all data are fully available without restriction

A Data Availability Statement describing where the data can be found is required at submission. Your answers to this question constitute the Data Availability Statement and **will be published in the article**, if accepted.<br><br>**Important:** Stating 'data available on request from the author' is not sufficient. If your data are only available upon request, select 'No' for the first question and explain your exceptional situation in the text box.<br><br>Do the authors confirm that all data underlying the findings described in their manuscript are fully available without restriction?	
**Describe where the data may be found in full sentences. If you are copying our sample text, replace any instances of XXX with the appropriate details.**<br><br>• If the data are **held or will be held in a public repository**, include URLs, accession numbers or DOIs. If this information will only be available after acceptance, indicate this by ticking the box below. For example: *All XXX files are available from the XXX database (accession number(s) XXX, XXX.).*<br>• If the data are all contained **within the manuscript and/or Supporting Information files**, enter the following: *All relevant data are within the manuscript and its Supporting Information files.*<br>• If neither of these applies but you are able to provide **details of access elsewhere**, with or without limitations, please do so. For example:<br><br>*Data cannot be shared publicly because of [XXX]. Data are available from the XXX Institutional Data Access / Ethics Committee (contact via XXX) for researchers who meet the criteria for access to confidential data.*<br><br>*The data underlying the results presented in the study are available from (include the name of the third party*	All relevant data are within the manuscript and its Supporting Information files.

and contact information or URL).<br>• This text is appropriate if the data are owned by a third party and authors do not have permission to share the data.<br><br>* typeset	
Additional data availability information:	Tick here if the URLs/accession numbers/DOIs will be available only after acceptance of the manuscript for publication so that we can ensure their inclusion before publication.

Powered by Editorial Manager® and ProduXion Manager® from Aries Systems Corporation

Dear editors,

We would like to submit the manuscript with the following title and authors for publication in ***Plos-One.***

Title: 1.25(OH)2D3 decrease PCNA and mTOR expression and alleviates renal injury in Thy-1 nephritis rat model

Authors: Jianfeng Li, Lei Jin, Huan Ma, Jie Suo, Rui Yang, Xiaoping Yang

Our results revealed that 1.25$(OH)_2D_3$ could significantly inhibit the proliferation of glomerular mesangial cells in rats. Also, mTOR protein was involved in the process of 1.25$(OH)_2D_3$ regulating glomerular mesangial cells. Our findings may provide new insights for understanding the molecular mechanism of 1.25$(OH)_2D_3$ in alleviating renal injury in glomerulonephritis.

We declare that submitted manuscript does not contain previously published material, and is not under consideration for publication elsewhere. The manuscript is truthful original work without fabrication, fraud or plagiarism. Each author has made an important scientific contribution to the study and is thoroughly familiar with the primary data. All authors listed have read the complete manuscript and have approved submission of the paper. All authors declare that there is no conflict interest.

This work was supported by the National Natural Resources Foundation of China (81160090) and Research Project Supported by Xinjiang Production and Construction Corps of China (2009GG59). The funders had no role in study design, data collection and analysis, decision to publish, or preparation of the manuscript.

We would be happy if the manuscript will be evaluated by your Editorial Board members for publication in ***Plos-One.***

With best regards,

Yours sincerely,

(Corresponding author)

Xiaoping Yang

Department of nephrology, The first affiliated hospital of Shi he zi University, Xin

jiang province, China

E-mail: xjshzdxyxp@163.com

Tel: +86-13579458071

Manuscript Click here to access/download;Manuscript;Manuscript.docx

$1.25(OH)_2D_3$ decreases PCNA and mTOR expression and alleviates renal injury in Thy-1 nephritis rat model

Jian-feng Li[1¶], Lei Jin[2¶],Huan Ma[3], Jie Suo[4], Rui Yang[5], Xiao-ping Yang[5,*]

1. Department of nephrology, The first people's hospital of Nanyang city, Henan province, 473000,China.
2. Department of Rheumatology, Immunology & Allergy, Shanghai General Hospital, Shanghai Jiao Tong University School of Medicine, Shanghai,200080, China.
3. Department of Gastrointestinal surgery, The first people's hospital of Nanyang city, Henan province, 473000, China.
4. Department of nephrology, The first people's hospital of Aksu city, Xin jiang province, 843000,China.
5. Department of nephrology, The first affiliated hospital of Shi he zi University, Xin jiang province, 832000,China.

¶These authors contributed equally to this work and share first authorship

***** Correspondence: xjshzdxyxp@163.com; Tel: +86-13579458071

Abbreviations

CG, Control Group

NG, Nephritis Group

mTOR, mammalian target of rapamycin

ESRD, end-stage renal disease

PI3K, phosphoinositide 3-kinase

HE, Hematoxylin and eosin

MPGN, mesangial proliferative glomerulonephritis

PCNA, proliferating cell nuclear antigen

Abstract

Objective: This study explored the role and mechanisms of 1.25$(OH)_2D_3$ in proliferative glomerulonephritis and its effect on the regulation of mesangial cells.

Methods: Sixty male SD rats were randomly divided into four groups: control (CG), nephritis (NG), nephritis + 1.25$(OH)_2D_3$ (NVG), and nephritis + 1.25$(OH)_2D_3$ + rapamycin (NVRG) (n=15 per group). Three rats from each group were sacrificed on days 1, 3, 7, 14, and 21 after intervention. Urine samples were collected from the rats over 24 hours on day 0 to measure urinary protein excretion. Renal tissue samples were stained with HE and PAS to assess the severity of renal injury, and immunohistochemistry was used to measure PCNA and mTOR expression in the renal tissues.

Results: Compared to NG, mesangial cell proliferation in the renal tissues was significantly alleviated in NVG and NVRG at all time points (all $p<0.05$); PCNA expression was significantly higher in the NG compared to the CG ($p < 0.05$) and significantly lower in the NVG and NVRG ($p < 0.05$). mTOR expression was also significantly increased in the NG compared to the CG, with a significant reduction observed in the NVG and NVRG compared to the NG.

Conclusion: Our findings clarify that 1.25$(OH)_2D_3$ could significantly inhibit the proliferation of glomerular mesangial cells in rats. Also, mTOR protein was involved in the process of 1.25$(OH)_2D_3$ regulating glomerular mesangial cells. Our resuits further elucidate the molecular mechanism of 1.25$(OH)_2D_3$ in alleviating renal injury in glomerulonephritis.

Keywords: 1.25$(OH)_2D_3$, mammalian target of rapamycin (mTOR), Thy-1 Nephritis,

Proliferation

Introduction

Glomerulonephritis is a common cause of end-stage renal disease (ESRD) [1]. Data from the National Dialysis Patient Registry of the China Renal Data System indicate that glomerulonephritis is the leading cause of ESRD in China [2, 3]. The key pathological features of glomerulonephritis include the proliferation of mesangial cells and abnormal deposition of extracellular matrix. Early treatment of patients with nephrotic syndrome using steroids can lead to better long-term remission [4]. However, in patients with membranous glomerulopathy or proliferative nephritis, steroids alone have shown only modest improvement in renal parameters [5]. Therefore, exploring novel therapeutic agents for treating mesangioproliferative glomerulonephritis and preventing ESRD is critically important.

1,25-Dihydroxyvitamin D_3 (1,25(OH)$_2D_3$) is the active form of vitamin D_3 and has been reported to possess anti-inflammatory, anti-tumor, and cardiovascular-protective properties [6-8]. Proper treatment can induce apoptosis in proliferated mesangial cells, allowing for the recovery of glomerular structure, as demonstrated in the Thy-1 nephritis model [9]. Inhibiting the proliferation of mesangial cells can delay disease progression and improve prognosis. Previous studies have shown that 1,25(OH)$_2D_3$ can significantly inhibit the proliferation of mesangial cells cultured in vitro and promote their differentiation and apoptosis [10, 11]. However, it remains unclear whether 1,25(OH)$_2D_3$ can regulate the pathology and progression of glomerulonephritis through mTOR.

mTOR has been reported to be involved in mesangial cell proliferation and oxidative damage in the development of chronic kidney diseases, such as diabetic nephropathy and IgA nephropathy [12, 13]. Activation of the phosphoinositide 3-kinase (PI3K)/protein kinase (Akt)/mammalian target of rapamycin (mTOR) signaling pathway can influence cell proliferation, hypertrophy, and apoptosis [14]. However, the role of the mTOR signaling

pathway in the regulation of mesangial cells by 1.25$(OH)_2D_3$ has been rarely explored.

In this study, we established Thy-1 rat nephritis models and administered 1.25$(OH)_2D_3$ and rapamycin to the experimental groups to observe changes in PCNA and mTOR expression in rat renal tissues at different time points.We also conducted a preliminary investigation into the target and mechanism of 1.25$(OH)_2D_3$ in regulating mesangial cells, aiming to provide a reliable theoretical basis for further research in this field.

Methods

Animals

Sixty male SD rats (6 weeks old, weighing 180-200 g) were obtained from the Xinjiang Center for Local Epidemic Disease Control. The rats were housed in an environment with a temperature of 22 ± 1 ºC, relative humidity of 50 ± 1%, and a 12/12-hour light/dark cycle, with noise levels below 85 dB. They were provided with food and water ad libitum. All animal procedures, including euthanasia, were approved by the Shihezi University Institutional Animal Care and Use Committee and were conducted in accordance with the regulations and animal welfare guidelines of Shihezi University (SCXK [Xin] 2013-178). The animals were randomly divided into four groups (15 rats per group): control group (CG), nephritis group (NG), nephritis + 1.25$(OH)_2D_3$ group (NVG), and nephritis + 1.25$(OH)_2D_3$ + rapamycin group (NVRG).

Thy-1 nephritis induction and treatment

Thy-1 nephritis models were prepared as previously described [15]. Briefly, rats in the NG, NVG, and NVRG groups received a single tail vein injection of 25 μL/100 g of Thy-1 monoclonal antibodies (Ontario, Canada, catalog No.: CL005A), while rats in the CG received an equivalent dose of saline. After injection, urine was collected from all groups over 24 hours using metabolic cages, and proteinuria in fresh urine was assessed using the standard Coomassie blue staining assay. After successful modeling, rats in the NVG received

a gastric perfusion of 0.5 μg/day of 1.25(OH) $_2$D$_3$ (Shanghai Roche Pharmaceuticals Ltd.) dissolved in 1 mL of peanut oil, and rats in the NVRG received a gastric perfusion of 0.5 μg/day of 1.25(OH) $_2$D$_3$ and 1 mg/day of rapamycin (Yixinke, Research Institute of North China Pharmaceutical Group) dissolved in 1 mL of peanut oil. Rats in the CG and NG were given a gastric perfusion of 1 mL/day of peanut oil. The Thy-1 nephritis model has a self-healing property, so observations were made on days 1, 3, 7, 14, and 21. On these days, 3 rats from each group were randomly sacrificed (using 2% pentobarbital sodium, 30 mg/kg, intraperitoneally), and kidney tissues were collected.

Hematoxylin and eosin (HE) staining and Periodic Acid-Schiff (PAS) staining

For HE staining, renal tissues were fixed in 10% formaldehyde, embedded in paraffin, and sectioned into 3-μm-thick slices. The sections were baked at 60°C for 2 hours, then placed in xylene and ethanol for dewaxing and hydration. They were stained with hematoxylin for 5 minutes and eosin for 3 minutes. For PAS staining, sections were treated with periodic acid, followed by washing and incubation with Schiff’s reagent, and then stained with hematoxylin. After dehydration with ethanol and xylene, the sections were sealed with neutral gum.

Pathological damage was graded according to the criteria for human mesangial proliferative glomerulonephritis (MPGN) as follows: grade 0, normal; grade I, mild mesangial proliferation; grade II, open capillary loops with mesangial width not exceeding the capillary diameter, showing segmental distribution, and moderate mesangial proliferation; grade III, compressed capillary loops with mesangial width exceeding the capillary diameter, showing diffuse distribution, severe proliferation, and severely compressed capillary loops, along with the presence of nodules and clumpy solid areas.

Immunohistochemistry

Immunohistochemistry was conducted using a universal two-step PV-9000 test kit (ZSGB-BIO, China) according to the manufacturer's instructions. PCNA antibodies (#2586, 1:50) and mTOR antibodies (#2983, 1:2000) were obtained from Cell Signaling Technology. Five fields of view at 400X magnification were randomly selected from each sample to assess the percentage of positive cells and staining intensity. The percentage of positive cells was scored as follows: 0 points for no staining, 1 point for<10% staining, 2 points for 10%-50% staining, and 3 points for>50% staining. Staining intensity was scored as follows: 0 points for no staining, 1 point for light brownish-yellow staining, 2 points for brownish-yellow staining, and 3 points for brown staining. The final score was calculated by multiplying the percentage of positive cells score by the staining intensity score: negative (-) with a final score of 0; weak positive (+) with a final score of 2-3; moderate positive (++) with a final score of 4-5; and strong positive (+++) with a final score of 7-9.

Statistical analysis

All data were presented as mean ± standard deviation and analyzed using SPSS software (version 13.0; SPSS, Inc.). A paired t-test was used for paired samples. A nonparametric test was employed to compare the expression levels of PCNA and mTOR proteins at different time points and among different groups. A P-value of <0.05 was considered statistically significant.

Results

1.25$(OH)_2D_3$ reduces proteinuria levels of Thy-1 nephritis rats

To evaluate whether 1.25$(OH)_2D_3$ can alleviate nephritis, proteinuria levels were measured in the CG, NG, NVG, and NVRG groups on days 0, 1, 3, 7,14, and 21. The amount

of water intake and urine volume across the four groups, as measured in metabolic cages, showed no statistically significant differences ($p>0.05$). Changes in urine protein levels are summarized in Table 1. Compared with the CG, the NG, NVG, and NVRG groups showed significantly higher proteinuria levels on day 1, confirming the successful establishment of the Thy-1 nephritis rat model. On days 3, 7, and 14, significant reductions in proteinuria were observed in the NVG and NVRG groups compared with the NG, indicating that 1.25(OH)$_2$D$_3$ and its combination with rapamycin had a significant ameliorating effect on Thy-1-induced nephritis (Figure 1, Table 1).

Table 1. Effect of 1.25(OH)$_2$D$_3$ treatment on proteinuria at different time points after the induction of glomerulonephritis

	day 0	Day 1	Day 3	Day 7	Day 14	Day 21
CG	1.517±0.712	1.119±0.434	1.528±0.107	2.748±0.569	1.769±0.174	2.54±0.439
NG	1.139±0.286	4.896±0.539*	10.98±0.156	8.04±1.699	4.45±0.354	2.482±0.594
NVG	0.786±0.181	3.015±0.397*	3.865±0.74#	5.53±1.057#	2.11±0.128#	2.241±0.688
NVRG	1.056±0.674	3.283±0.205*	3.157±0.205#	4.511±0.781#	1.808±0.593#	1.463±0.879

Note: *$p<0.05$, compared to CG, indicates significant higher proteinuria on Day 1 after antibody injection. *$p<0.05$, compared to CG, indicates significantly decreased mean urinary protein level of NVG on Day 3, Day 7, and Day 14. Unit: mg/d

1.25(OH)$_2$D$_3$ relieves renal injury in the Thy-1 induced nephritis rat model

The effect of 1.25(OH)$_2$D$_3$ on renal pathology was further evaluated using HE and PAS staining (Figures 2-3). The renal tissues of the CG appeared normal, with no proliferation of mesangial cells or extracellular matrix, resulting in a grade 0 for pathological damage. In contrast, the NG showed significant mesangial cell proliferation, severe compression of capillary loops, nodules, and clumpy solid areas at all time points, with the most severe

pathological injury observed on day 7, which then showed some alleviation by day 21. Additionally, some rats in the NG exhibited glomerular lobulation, sclerosis, and fibrosis. Compared to the NG, mesangial cell proliferation in the NVG and NVRG groups was significantly reduced at all time points (all $p<0.05$), particularly on days 7 and 14. The capillary loops in these groups were only mildly compressed, with the mesangial width not exceeding the capillary diameter and showing a segmental distribution (Table 2). These findings suggest that $1.25(OH)_2D_3$ reduces renal injury, including mesangial cell proliferation.

Table 2. Comparison of pathological damage grading of renal tissues among different groups at all time points

	Day 1	Day 3	Day 7	Day 14	Day21
CG	0.00±0.000	0.00±0.000	0.00±0.000	0.00±0.000	0.00±0.000
NG	0.67±0.516	1.33±0.516*	2.83±0.408*	2.00±0.632	0.83±0.754
NVG	0.83±0.408	1.00±0.632*	1.83±0.752#	1.17±0.408#	0.67±0.516
NVRG	0.67±0.516	0.83±0.753*	1.83±0.408#	1.00±0.632#	0.67±0.516

Note: $^{*}p<0.05$, indicates the severe renal pathological damage on compared to CG from Day 3. $^{\#}p<0.05$, indicates significantly alleviated pathological renal damage of NVG and NVRG on Day 7 and Day 14 compared to NG.

PCNA and mTOR expressions measured by immunohistochemistry

The results above indicate that $1.25(OH)_2D_3$ can suppress proteinuria and reduce mesangial cell proliferation and abnormal extracellular matrix deposition, demonstrating its protective effects on renal function. To investigate the underlying mechanism, we performed

immunohistochemistry to assess PCNA and mTOR expression. PCNA expression in mesangial cells appeared as brown-yellow granules located in the nucleus, occasionally observed in glomerular mesangial cells of normal rats. The quantification of PCNA expression is summarized in Table 3. Compared with the CG, the NG showed a significantly increased level of PCNA expression in renal tissues ($p<0.05$) and in glomerular mesangial cells on days 1, 3, 7, 14, and 21. The PCNA expression in the NG increased over time, peaking on day 7, which aligns with the trend in mesangial cell proliferation. Compared to the NG, both the NVG and NVRG groups exhibited significantly lower PCNA expression in glomerular mesangial cells at all time points ($p<0.05$), with a reduction in PCNA expression observed on days 14 and 21 compared to day 7 (Figure 4) ($p<0.05$).

mTOR expression in mesangial cells was visualized as nest-like, cord-like, or diffusely distributed brown-yellow granules located in the nucleus. The expression levels of mTOR across different groups and time points are summarized in Table 4. In the NG, mTOR expression was significantly higher in renal tissues and glomerular mesangial cells compared to the CG ($p<0.05$). In the CG, there were no significant changes in mTOR expression across the different time points (all $p>0.05$). However, in the NVG and NVRG groups, mTOR expression was significantly lower compared to the NG at all time points ($p<0.05$), with mTOR expression levels decreasing over time (Figure 5).

Table 3. Comparison of PCNA expression grading among different groups at different time points

	day 1	day 3	day 7	day 14	day 21
CG	0.00±0.00●	0.00±0.00	0.00±0.00▲	0.00±0.00	0.00±0.00
NG	3.41±1.23●	5.44±2.09	8.14±2.65▲	6.58±1.86	2.92±1.51
NVG■	3.62±2.97	4.33±0.67	4.81±1.16★	4.42±1.56	2.47±1.17
NVRG■	2.57±1.07	3.53±1.39	5.27±2.18※	4.75±2.17	2.11±1.63

Note: indicates PCNA expression in renal tissues on Day 1 after antibody injection and ●

$p<0.01$; indicates the highest PCNA expression in renal tissues on Day 7 after antibody injection and ▲$p<0.01$; indicates a lower PCNA expression of NVG than that of NG on Day 7 and ★$p<0.05$; ※ indicates a decreased PCNA expression of NVRG on Day 7 and $p<0.05$; indicates no significant difference in PCNA expression between NVG and NVRG at all time points and ■$p>0.05$.

Table 4. Comparison of mTOR expression grading among different groups at different time points

	day 1	day 3	day 7	day 14	day 21
CG	1.33±0.58●	2.00±2.00	1.33±2.31▲	2.00±2.00	2.33±1.52
NG	6.00±3.00●	8.00±1.00	12.00±0.00▲	8.00±1.00	3.33±1.15
NVG■	4.67±1.15	4.67±1.15	10.00±1.00★	3.33±1.15	2.33±1.15
NVRG■	3.81±1.15	3.91±2.31	10.00±3.00※	4.46±1.15	3.00±2.00

Note: indicates mTOR expression in renal tissues on Day 1 after antibody injection and •$p<0.01$; indicates the highest mTOR expression in renal tissues on Day 7 after antibody injection and ▲$p<0.01$; indicates a lower mTOR expression of NVG than that of NG on Day 7 and ★$p<0.05$; ※ indicates a decreased mTOR expression of NVRG on Day 7 and $p<0.05$; indicates no significant difference in mTOR expression between NVG and NVRG at all time points and ■ $p>0.05$.

The expression of PCNA expression and mTOR is associated with a degree of renal pathological in Thy-1 nephritis rats

The relationship between PCNA or mTOR expression and renal pathological damage was analyzed, revealing that the 24-hour urinary protein levels, PCNA expression, and mTOR expression were positively correlated with each other ($p<0.05$), and all were positively correlated with the pathological damage grading ($p<0.05$), as shown in Table 5.

Table 5. Correlation among pathological damage grading, PCNA expression, and mTOR

expression in glomerular renal tissues

	r	P
Pathological damage and mTOR	0.729	P<0.001
Pathological damage and PCNA	0.652	P<0.001
PCNA and mTOR	0.677	P<0.001

Discussion

Abnormal proliferation of mesangial cells, which are key intrinsic cells of the glomerulus, along with the release of inflammatory mediators and pathological accumulation of extracellular matrices, are critical factors leading to glomerular sclerosis and the progression of various glomerulonephritides to end-stage renal disease [16, 17]. Therefore, developing new drugs and therapeutic strategies for glomerulonephritis that maintain a balance between mesangial cell proliferation, hypertrophy, and apoptosis, while improving the metabolism of mesangial matrices, is essential to delay or reverse glomerular sclerosis. Previous studies have shown that 1.25(OH) $_2D_3$ can inhibit mesangial cell proliferation and induce apoptosis by blocking the cell cycle [18, 19]; however, the underlying mechanism remains unclear. In this study, we established a nephritis rat model induced by Thy-1 injection and evaluated renal pathology to determine the roles and underlying mechanisms of 1.25(OH) $_2D_3$ in glomerulonephritis.

Compared with the CG, the experimental groups developed significant proteinuria on day 1 after antibody injection, which peaked on day 3 and returned to normal levels by day 14, likely due to the self-healing nature of the Thy-1 nephritis model. The NVG showed lower urinary protein levels than the NG at all time points, indicating that 1.25(OH) $_2D_3$ could reduce proteinuria and protect the kidneys, consistent with the findings of Szeto CC [14]. Additionally, the NVG exhibited significantly milder histopathological changes compared to the NG at all time points, including reduced mesangial cell proliferation, decreased

extracellular matrix accumulation, alleviated inflammatory factor buildup, and fewer balloon adhesions observed on day 7. These findings suggest that 1.25(OH) $_2D_3$ mitigates Thy-1 nephritis not only by reducing proteinuria but also by inducing favorable histopathological changes.

mTOR plays a key role in cell survival, proliferation, and apoptosis [20]. It is often continuously activated in many tumor cells, indicating its close relationship with the proliferation, survival, and metastasis of these cells [21]. A recent study demonstrated that mTOR is upregulated in mice with lupus nephritis and is strongly associated with the pathological proliferation of mesangial cells [10]. 1.25(OH) $_2D_3$ has been shown to induce differentiation and inhibit cell proliferation in various tissues. However, whether mTOR mediates this biological effect and whether there is a correlation between them remains unclear. In a 2006 study, Regulska et al. found that mTOR is involved in the protection of nerve cells by 1.25(OH) $_2D_3$ and its analogs [22], as well as in the inhibition of staurosporine-mediated apoptosis of osteoblasts by 1.25(OH) $_2D_3$ and its receptors [23]. Currently, there are few reports on the relationship between mTOR and mesangial cell proliferation. In this study, mTOR expression in the experimental groups increased from day 1, peaked on day 7, and then gradually declined. Both NVG and NVRG had lower mTOR expression levels compared to NG at all time points ($p<0.05$), but there was no statistically significant difference between NVG and NVRG ($p>0.05$). The expression of mTOR was positively correlated with the expression of PCNA, a cell cycle regulatory protein, suggesting that 1.25(OH) $_2D_3$ may inhibit mTOR expression and, thus, the proliferation of mesangial cells. We also observed that the combined intervention of rapamycin and 1.25(OH) $_2D_3$ did not produce a synergistic effect in rats with nephritis. There was no significant difference in mTOR expression between rats treated with both agents and those treated with 1.25(OH) $_2D_3$ alone, suggesting that rapamycin and 1.25(OH) $_2D_3$ may share the same target, the mTOR

protein. The specific mechanism by which 1.25(OH) $_2D_3$ inhibits mTOR expression should be explored further from a molecular biology perspective.

Our comprehensive analysis of the effects of 1.25(OH) $_2D_3$ on renal histopathology, mTOR protein, and PCNA in rat nephritis models showed that the grading of pathological renal damage, PCNA expression, and mTOR expression were positively correlated ($p<0.05$). These findings suggest that mTOR and PCNA may be involved in the inhibition of mesangial cell proliferation and the alleviation of renal injury by 1.25(OH) $_2D_3$.

The present study has some limitations, as we did not explore the possible molecular mechanisms in greater depth. In our future research, we will conduct PCNA and mTOR knockdown experiments to further validate their roles in the protective effects of 1.25(OH) $_2D_3$ against glomerulonephritis.Additionally, only a single dose of 1.25(OH) $_2D_3$ was used in this study, so different doses will be investigated in future studies.

In conclusion, our results suggest that 1.25(OH) $_2D_3$ significantly inhibits mesangial cell proliferation in rats, potentially through the reduction of mTOR and PCNA expression in nephrotic rats. The mTOR protein may be a key target of the biological effects of 1.25(OH) $_2D_3$.

Acknowledgments

Not applicable.

FundingThis work was supported by the National Natural Resources Foundation of China (81160090) and the Research Project Supported by Xinjiang Production and Construction Corps of China (2009GG59). The funders had no role in study design, data collection and analysis, decision to publish, or preparation of the manuscript.

Availability of data and materials

All data generated or analyzed during this study are included in this article.

Authors' contributions

All authors contributed to the study's conception and design. Xiao-ping Yang conceived and supervised the study; Xiao-ping Yang and Jian-feng Li designed the experiments; Jian-feng Li and Jie Suo performed the experiments; Lei Jin and Huan Ma analysed the data; Jian-feng Li and Lei Jin wrote the manuscript; Xiaoping Yang and Rui Yang revised the manuscript. All authors reviewed the results and approved the final version of the manuscript.

Consent to Participate

Not applicable.

Consent to Publish

Not applicable.

Competing interests

The authors have no relevant financial or non-financial interests to disclose.

Financial interests

The authors declare they have no financial interests.

References

1. Wang F, Yang C, Long J, et al. Executive summary for the 2015 Annual Data Report of the China Kidney Disease Network (CK-NET). Kidney international. 2019;95(3): 501-505. PMID: 30784660

2. Wei X, Li Z, Chen W, et al. Prevalence and risk factors of chronic kidney disease in first-degree relatives of chronic kidney disease patients in Southern China. Nephrology (Carlton, Vic).2012; 17(2): 123-130. PMID: 21919998

3. Liu ZH. Nephrology in china. Nature reviews Nephrol. 2013;9(9): 523-528. PMID: 23877587

4. Lieberman KV ,Pavlova-Wolf A. Adrenocorticotropic hormone therapy for the treatment of idiopathic nephrotic syndrome in children and young adults: a systematic review of early clinical studies with contemporary relevance. Journal of nephrology. 2017;30(1): 35-44. PMID: 27084801

5. Heidy Hendra ,Alan D Salama. Steroids as treatment for glomerulonephritis: time for a rethink. Nephrology, dialysis, transplantation : official publication of the European Dialysis and Transplant Association - European Renal Association . 2022;37(7): 1212-1217. PMID: 33188694

6. Ayse Sagmak Tartar , Safak Ozer Balin .Geriatric urinary tract infections: The value of laboratory parameters in estimating the need for bacteremia and Intensive Care Unit. Pakistan journal of medical sciences. 2019;35(1): 215-219. PMID: 30881426

7. Han J, Tang Y, Zhong M and Wu W.Antitumor effects and mechanisms of 1,25(OH)2D3 in the Pfeiffer diffuse large B lymphoma cell line. Molecular medicine reports. 2019;20(6): 5064-5074. PMID: 31638226

8. Da J, Yang Y, Dong R, Shen Y and Zha Y. Therapeutic effect of 1,25(OH)2-VitaminD3 on fibrosis and angiogenesis of peritoneum induced by chlorhexidine. Biomedicine & pharmacotherapy. 2020;129: 110431. PMID: 32585450

9. Hughes J, Savill JS. Apoptosis in glomerulonephritis. Current opinion in nephrology and hypertension. 2004;30(3): 655-676. PMID: 15261347

10. Abe H, Iehara N, Utsunomiya K, Kita T and Doi T.A vitamin D analog regulates mesangial cell smooth muscle phenotypes in a transforming growth factor-beta type II receptor-mediated manner. The Journal of biological chemistry. 1999;274(30): 20874-20878. PMID: 10409630

11. Weinreich T, Merke J, Schönermark M, et al. Actions of 1,25-dihydroxyvitamin D3 on human mesangial cells. American journal of kidney diseases. 1991;18(3): 359-366. PMID: 1652888

12. Han F, Xue M, Chang Y, et al. Triptolide Suppresses Glomerular Mesangial Cell Proliferation in Diabetic Nephropathy Is Associated with Inhibition of PDK1/Akt/mTOR Pathway. International journal of biological sciences. 2017;13(10): 1266-1275. PMID: 29104493

13. Xia M, Liu D, Tang X, et al. Dihydroartemisinin inhibits the proliferation of IgAN mesangial cells through the mTOR signaling pathway. International immunopharmacology. 2020;80: 106125. PMID: 31931362

14. LoPiccolo J, Blumenthal GM, Bernstein WB and Dennis PA. Targeting the PI3K/Akt/mTOR pathway: effective combinations and clinical considerations. Drug resistance updates : reviews and commentaries in antimicrobial and anticancer chemotherapy. 2008;11(1-2): 32-50. PMID: 18166498

15. Hashimoto K, Kamijo Y, Nakajima T, et al. PPARα Activation Protects against Anti-Thy1 Nephritis by Suppressing Glomerular NF-κB Signaling. PPAR research . 2012;2012: 976089. PMID: 22675338

16. Lei D, Chengcheng L, Xuan Q, et al. Quercetin inhibited mesangial cell proliferation of early diabetic nephropathy through the Hippo pathway. Pharmacological research. 2019;146: 104320. PMID: 31220559

17. Liu L, Ge W, Zhang Z, et al. Sublytic C5b-9 triggers glomerular mesangial cell proliferation via enhancing FGF1 and PDGFα gene transcription mediated by GCN5-dependent SOX9 acetylation in rat Thy-1 nephritis. FASEB journal : official publication of the Federation of American Societies for Experimental Biology. 2021;35(7): e21751. PMID: 34156114

18. Zhang CJ, Zhao D, Yin X, et al. Effects of 1,25(OH)2D3 on proliferation and apoptosis of human glomerular mesangial cells. American journal of translational research. 2016;8(6): 2659-2666. PMID: 27398149

19. Panichi V, Migliori M, Taccola D, et al. Effects of 1,25(OH)2D3 in experimental mesangial proliferative nephritis in rats. Kidney international. 2001;60(1): 87-95. PMID: 11422740

20. Suto T and Karonitsch T. The immunobiology of mTOR in autoimmunity. Journal of autoimmunity. 2020;110: 102373. PMID: 31831256

21. Murugan AK. mTOR: Role in cancer, metastasis and drug resistance. Seminars in cancer biology. 2019; 59: 92-111. PMID: 31408724

22. Zhang C, Chan CCY, Cheung KF, et al. Effect of mycophenolate and rapamycin on renal fibrosis in lupus nephritis. Clin Sci (Lond). 2019;133(15):1721-1744. PMID: 31358596

23.Regulska M, Leśkiewicz M, Budziszewska B, et al. Inhibitory effects of 1,25-dihydroxyvitamin D3 and its low-calcemic analogues on staurosporine-induced apoptosis. Pharmacological reports : PR. 2007;59(4): 393-401. PMID: 17901567

24. Zhang X and Zanello LP. Vitamin D receptor-dependent 1 alpha,25(OH)2 vitamin D3-induced anti-apoptotic PI3K/AKT signaling in osteoblasts. Journal of bone and mineral research : the official journal of the American Society for Bone and Mineral Research. 2008; 23: 1238-1248. PMID: 18410228

Figure legends

Figure 1 Measurement of proteinuria levels in CG, NG, NVG, and NVRG.

Figure 2HE staining images of renal tissues (×400).

Figure 3PAS staining images of pathological changes in renal tissues among 4 groups (×400).

Figure 4 Representative immunohistochemistry images of PCNA expression in renal tissues among CG, NG, NVG, and NVRG (×400).

Figure5 Representative immunohistochemistry images of mTOR expression in renal tissues among CG, NG, NVG, NVRG (×400).

Figure

Click here to access/download;Figure;Figure 1.tif

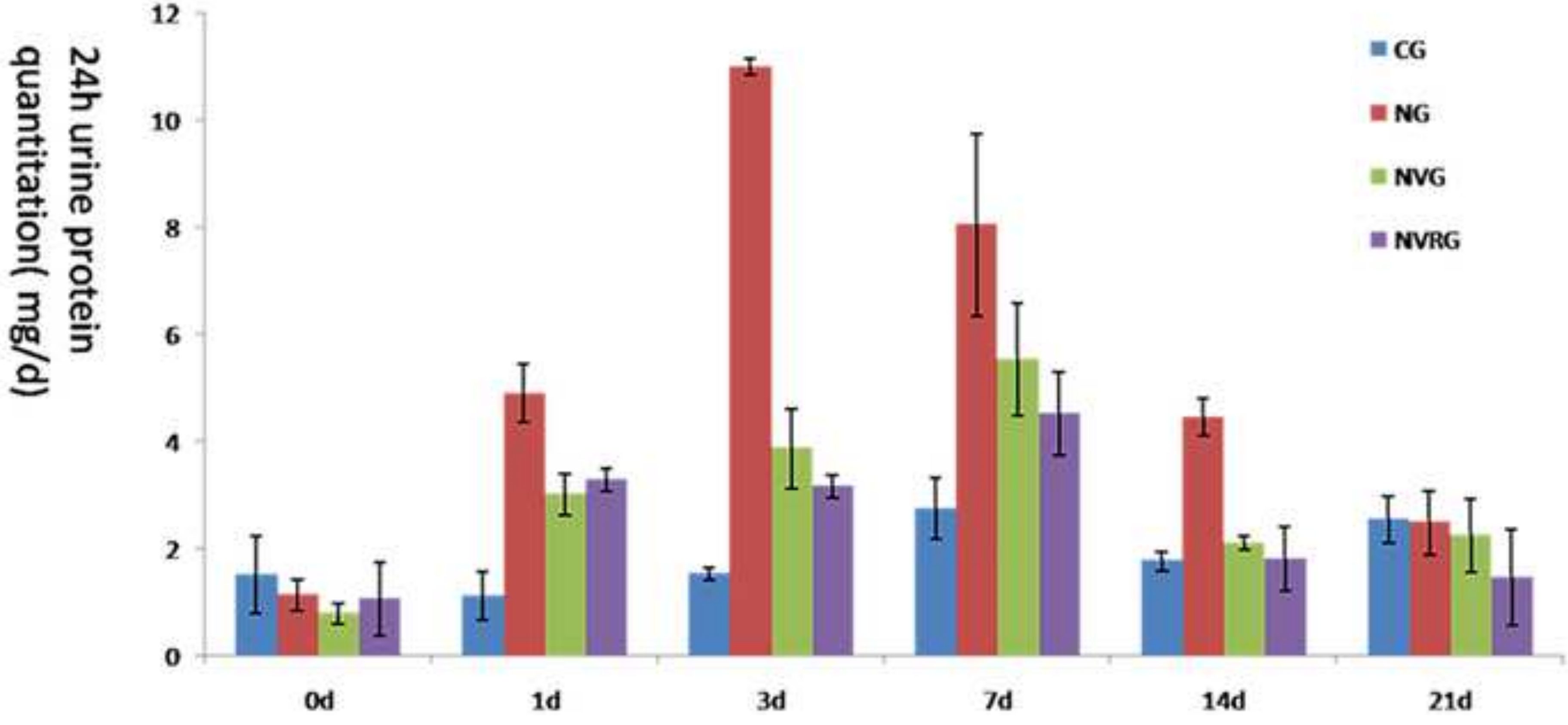

Click here to access/download;Figure;Figure 2.tif

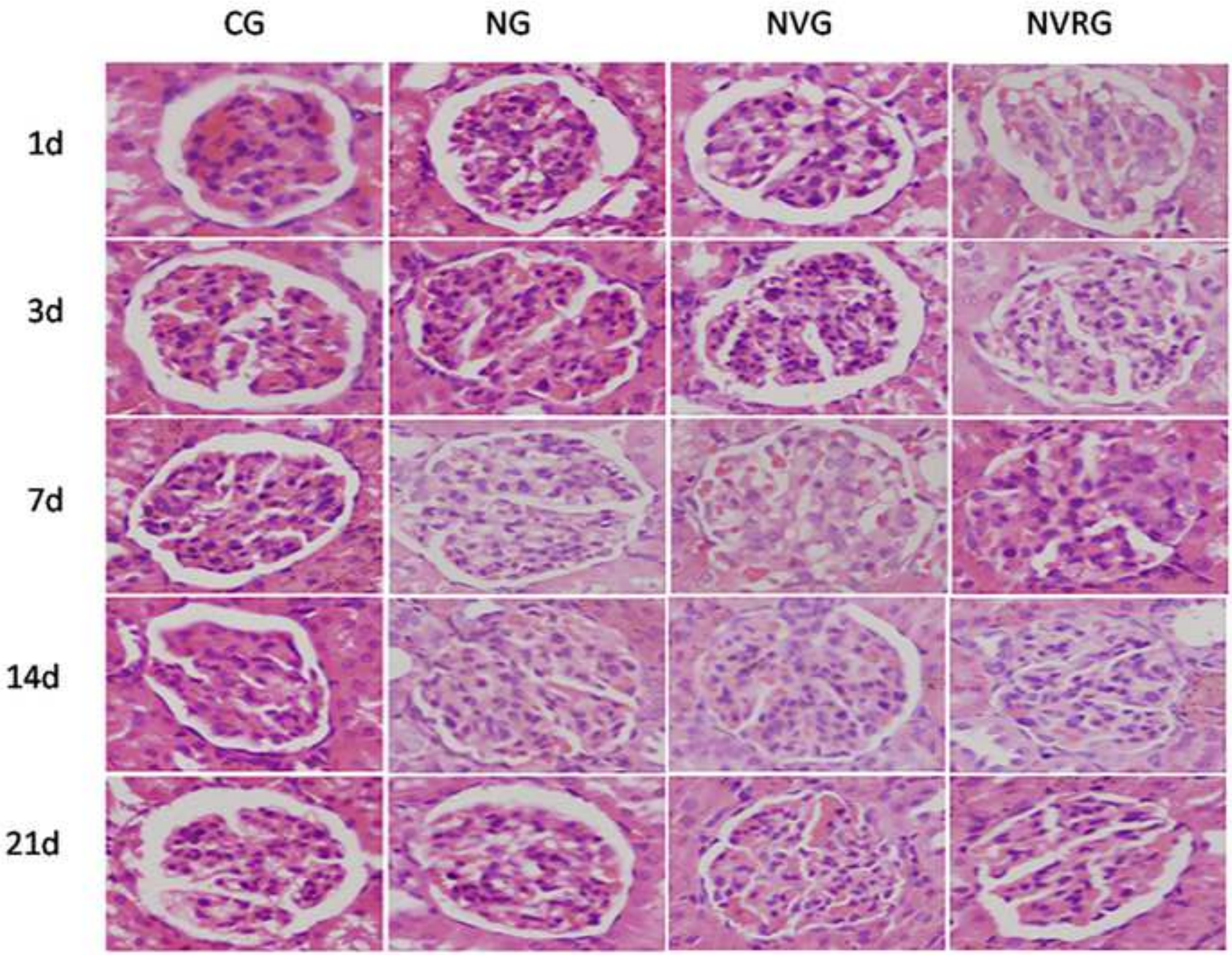

 Click here to access/download;Figure;Figure 3.tif

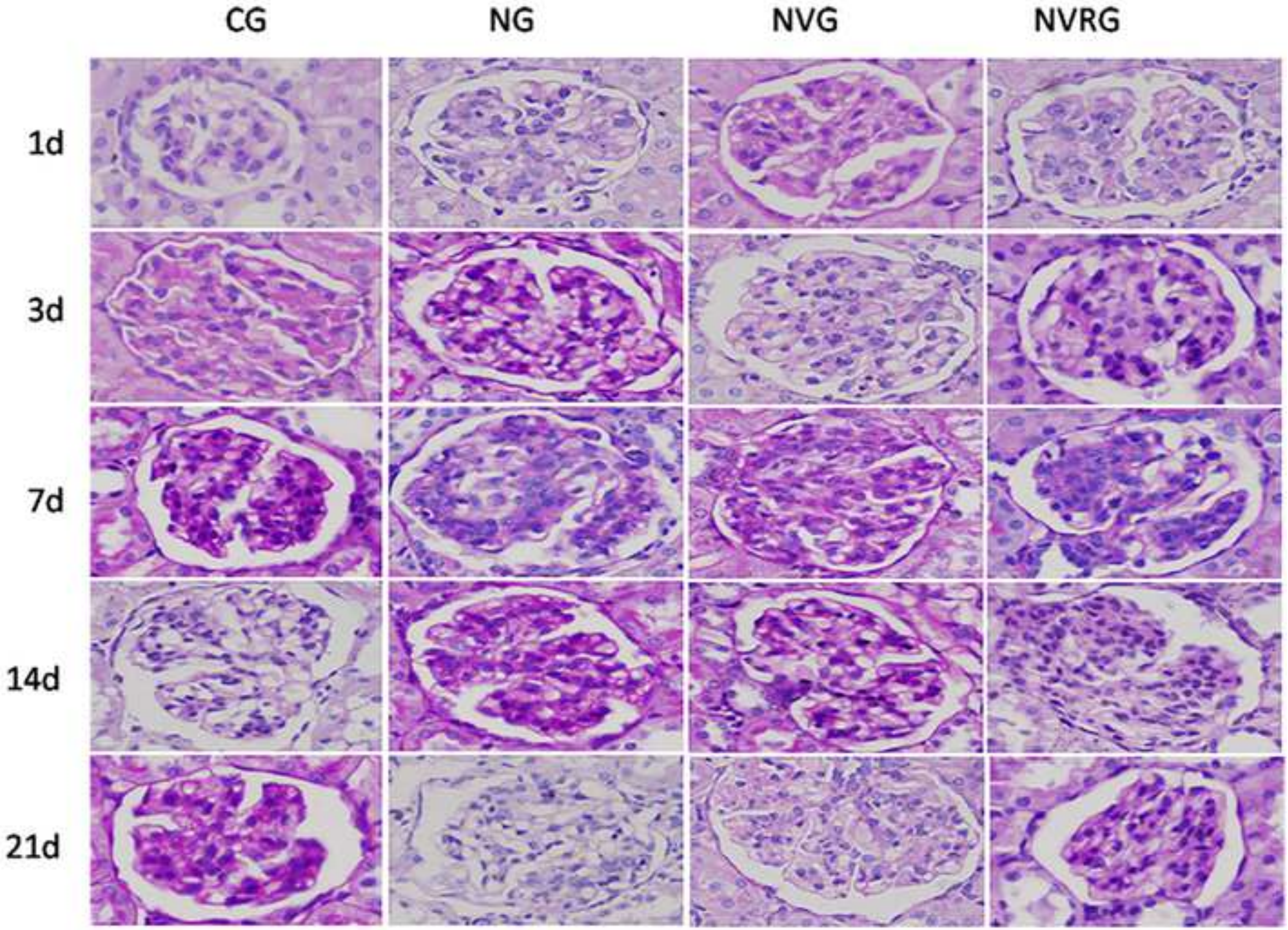

Click here to access/download;Figure;Figure 4.tif

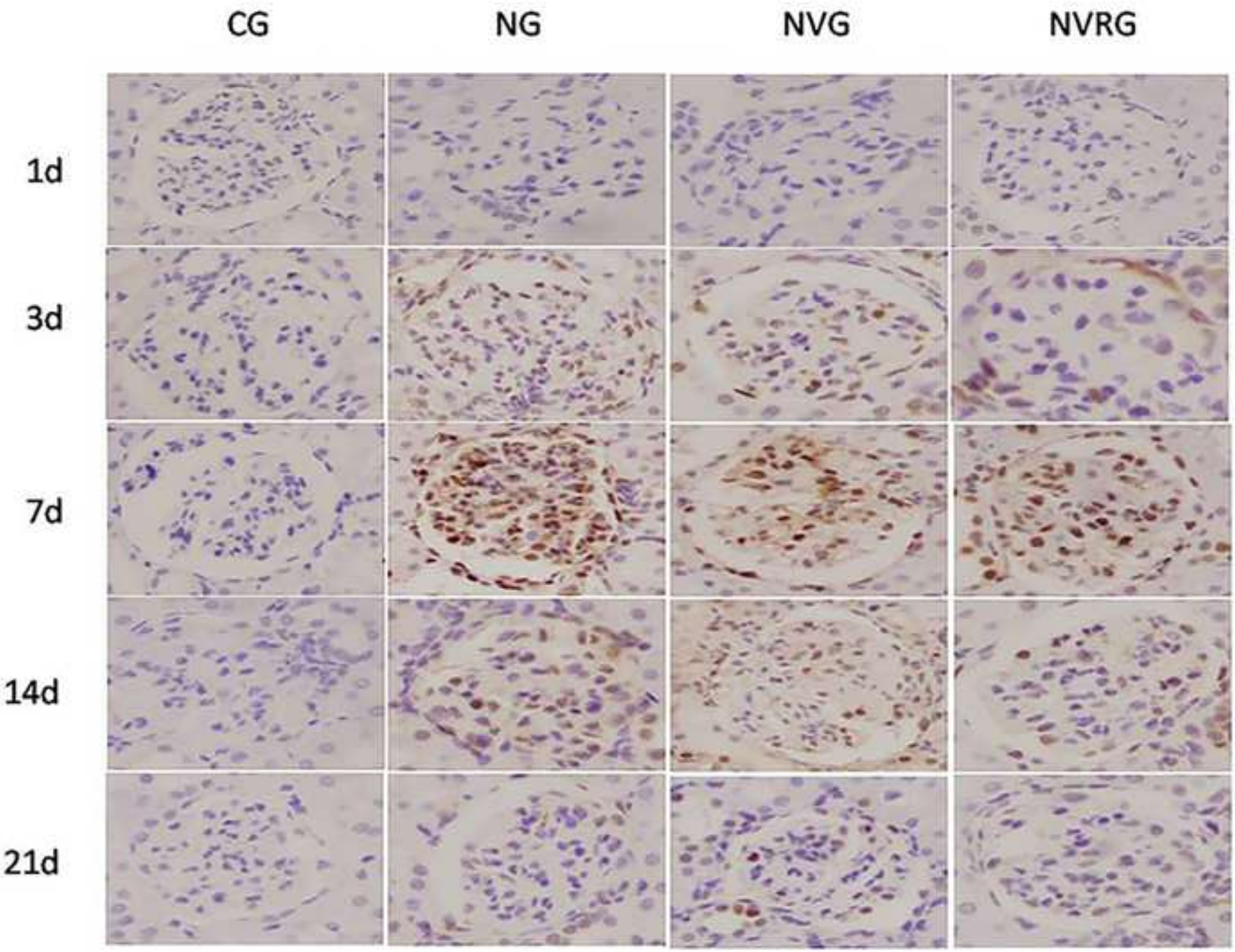

Figure

Click here to access/download;Figure;Figure 5.tif

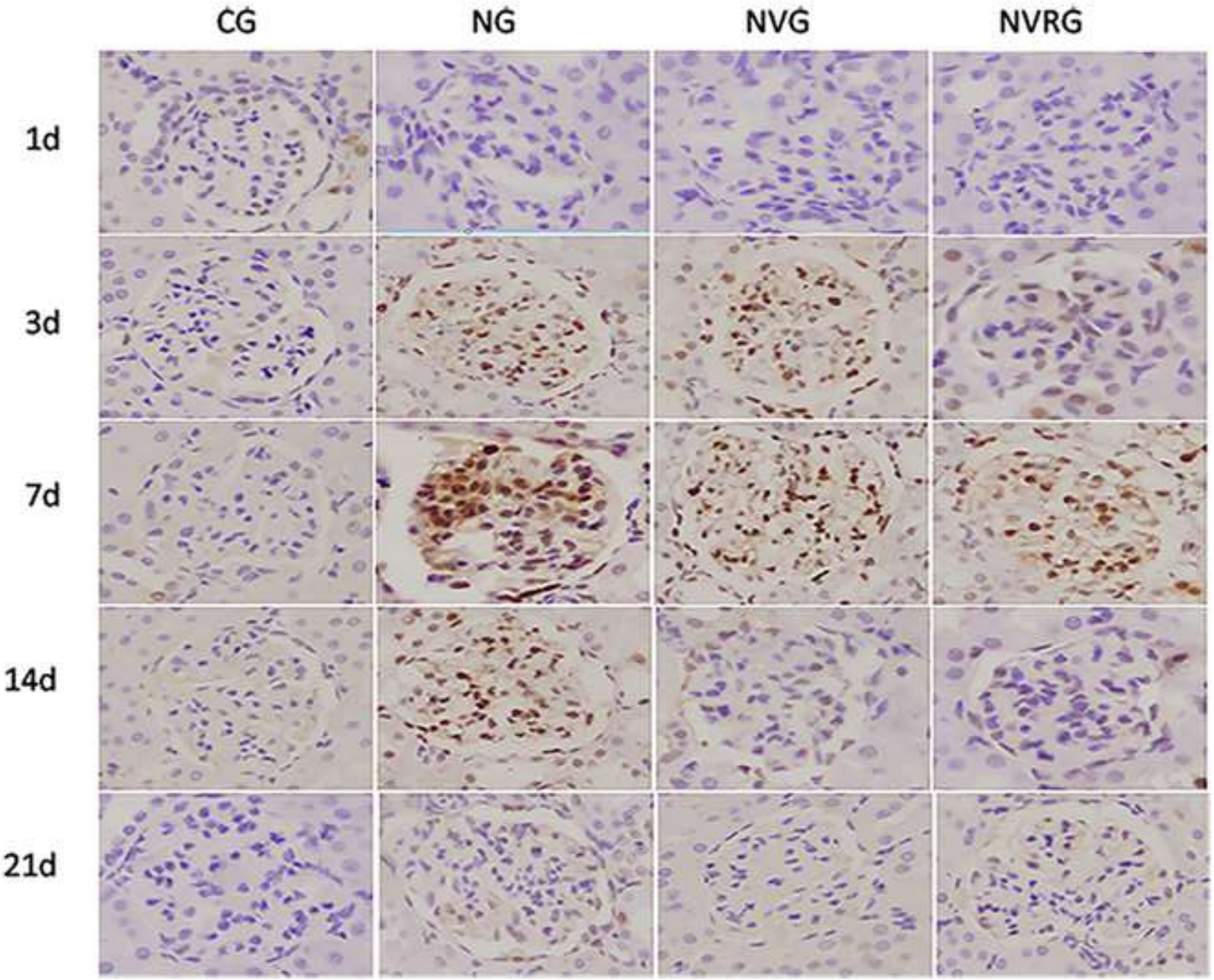

Figure 1 Measurement of proteinuria levels in CG, NG, NVG, and NVRG.

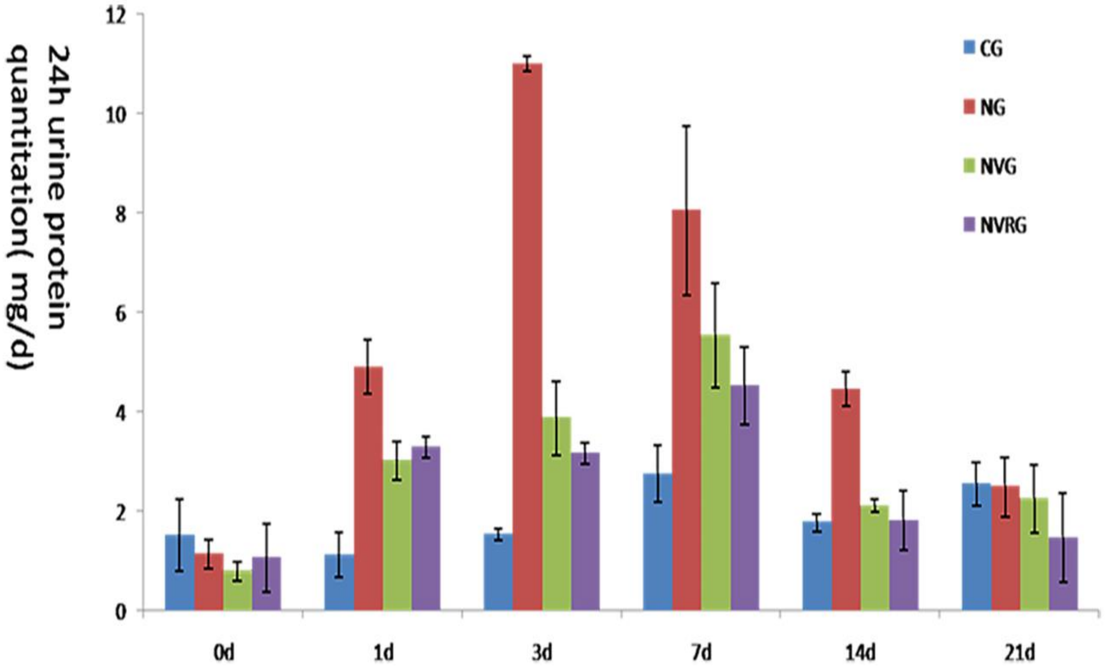

Figure 2 HE staining images of renal tissues (×400).

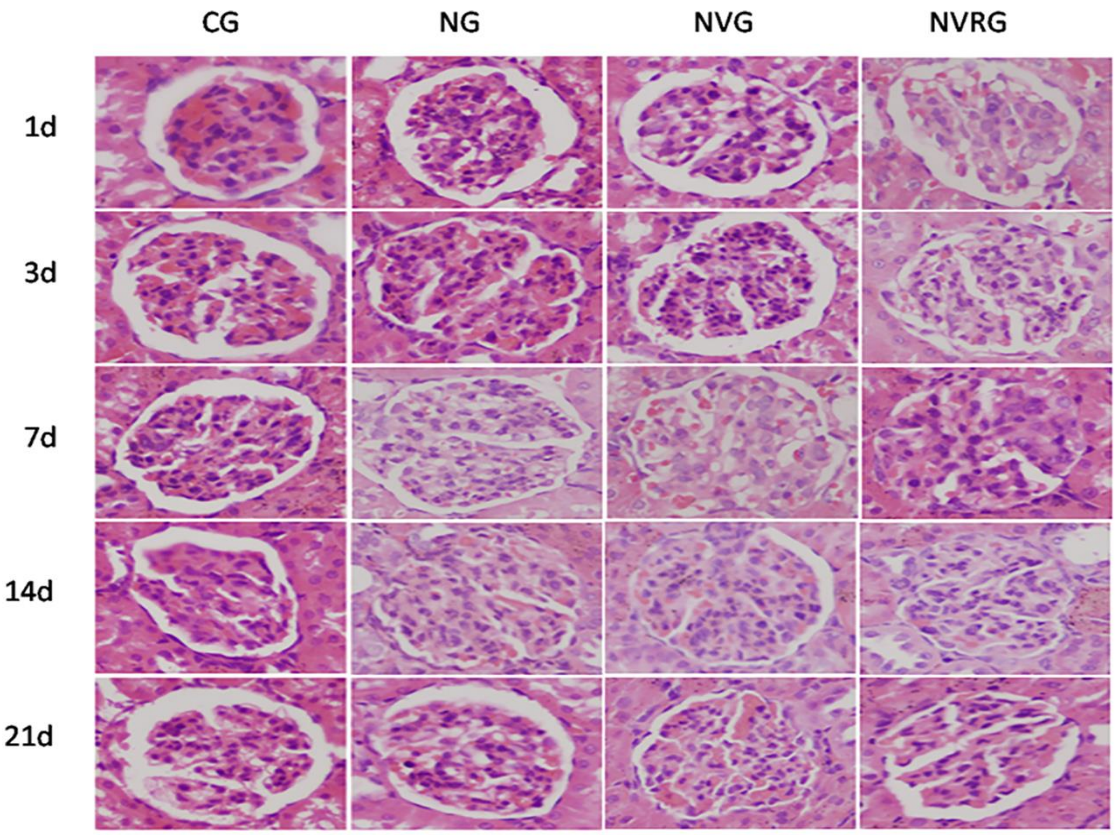

Figure 3 PAS staining images of pathological changes in renal tissues among 4 groups (×400).

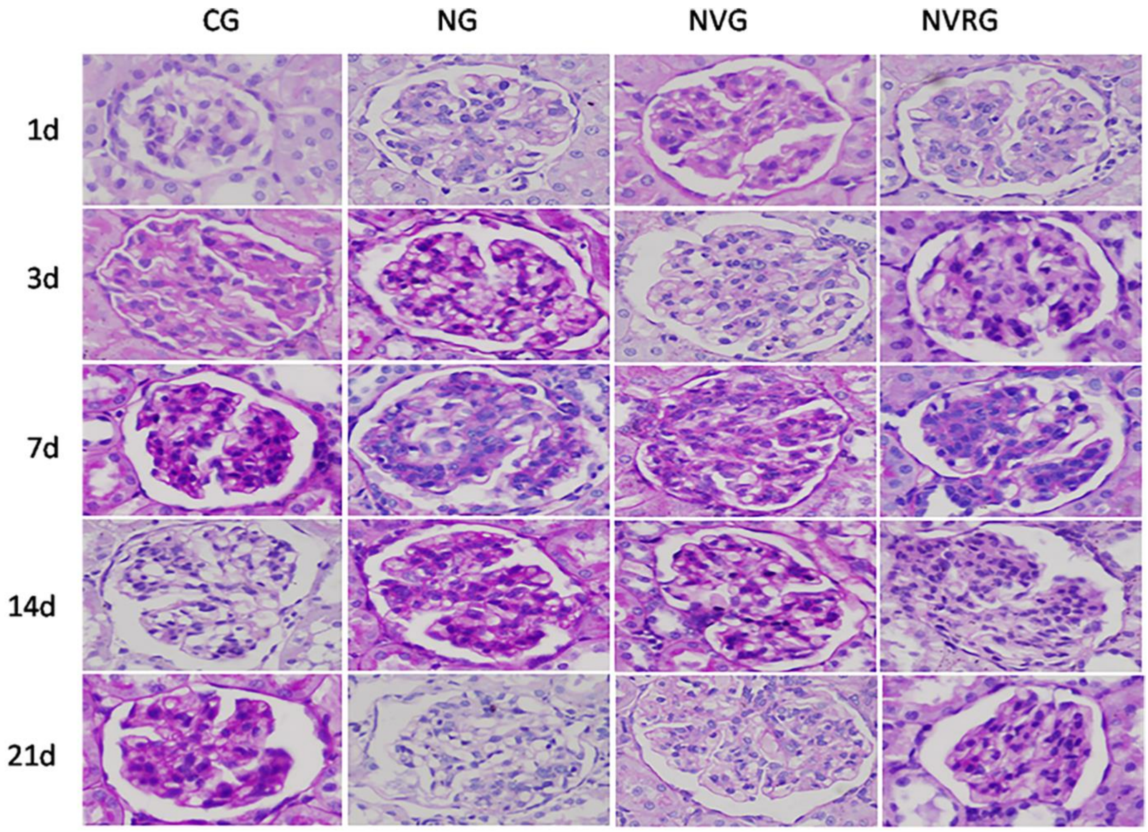

Figure 4 Representative immunohistochemistry images of PCNA expression in renal tissues among CG, NG, NVG, and NVRG (×400).

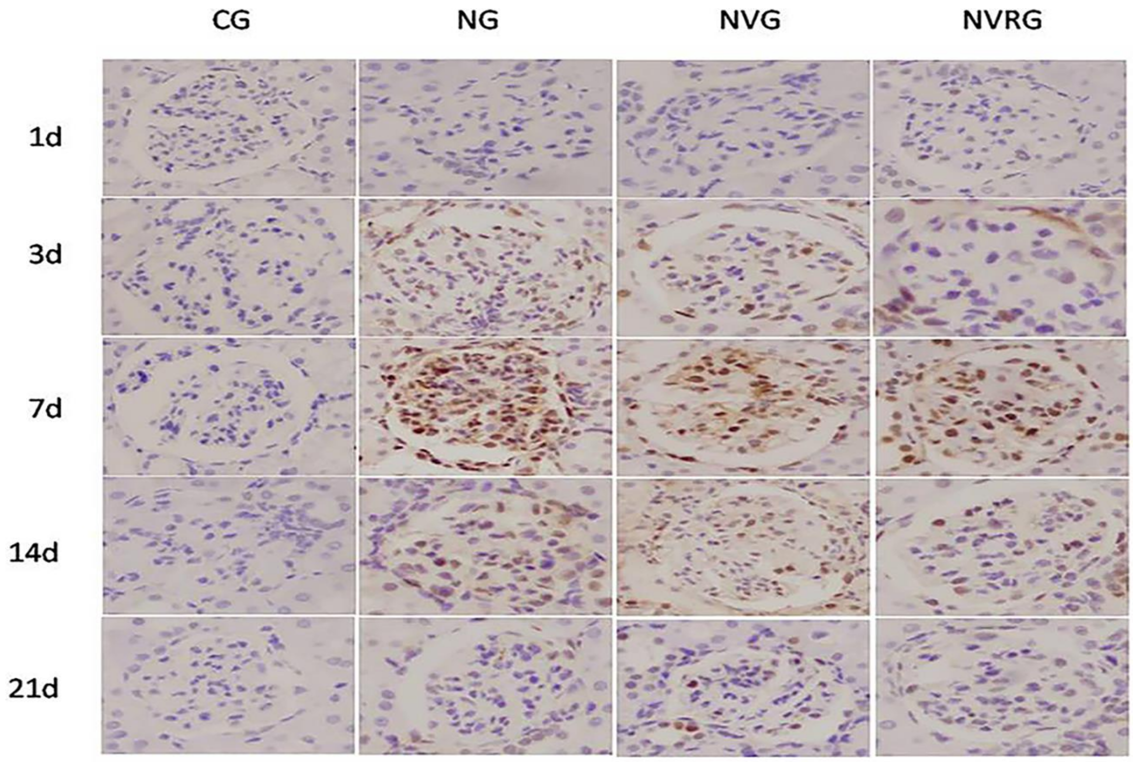

Figure 5 Representative immunohistochemistry images of mTOR expression in renal tissues among CG, NG, NVG, NVRG (×400).

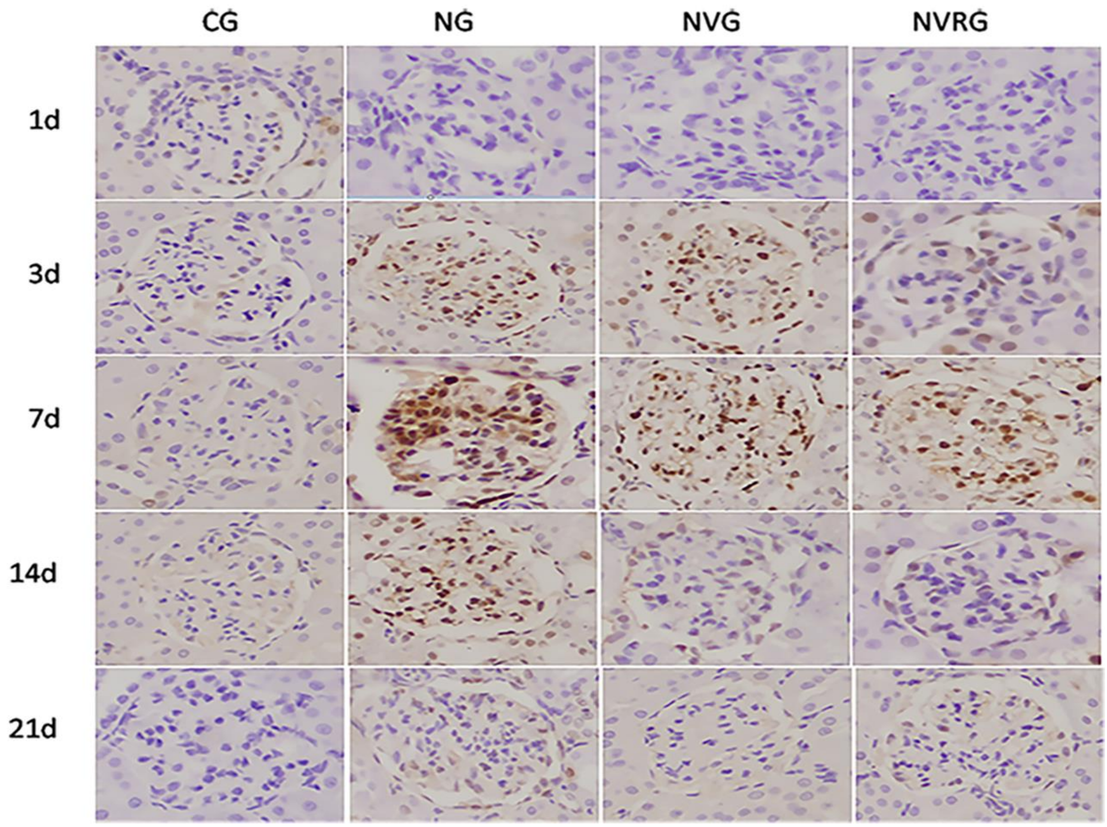

Click here to access/download
Other
English certificate.pdf

Other

Click here to access/download
Other
original data.xlsx

Click here to access/download
Other
Response for Journal requirements .docx

1.25(OH)~~2D3~~$_2$D$_3$ decreases PCNA and mTOR expression and alleviates renal injury in Thy-1 nephritis rat model

Jian-feng Li[1¶][~~1, #~~], Lei Jin[2¶][~~2, #~~],Huan Ma[3], Jie Suo[4], Rui Yang[5], Xiao-ping Yang[5,*]

1. Department of nephrology, The first people’s hospital of Nanyang city, Henan province, 473000,China.
2. Department of Rheumatology, Immunology & Allergy, Shanghai General Hospital, Shanghai Jiao Tong University School of Medicine, Shanghai 200080, China.
3. Department of Gastrointestinal surgery, The first people’s hospital of Nanyang city, Henan province,473000, China.
4. Department of nephrology, The first people’s hospital of Aksu city, Xin jiang province, 843000,China.
5. Department of nephrology, The first affiliated hospital of Shi he zi University, Xin jiang province,832000, China.

[¶#]These authors contributed equally to this work and share first authorship

***** Correspondence: xjshzdxyxp@163.com; Tel: +86-~~18336625063~~13579458071

Abbreviations

CG, Control Group

NG, Nephritis Group

mTOR, mammalian target of rapamycin

ESRD, end-stage renal disease

PI3K, phosphoinositide 3-kinase

HE, Hematoxylin and eosin

MPGN, mesangial proliferative glomerulonephritis

PCNA, proliferating cell nuclear antigen

Abstract

Objective: ~~The present studyexplored~~This study explored the ~~roleand~~role and mechanisms ~~of1~~of 1.25(OH~~)2D3~~)$_2$D$_3$ in ~~thylakoid~~ proliferative glomerulonephritis and its effect on the ~~modulation~~regulation of ~~thylakoid~~mesangial cells.

Methods: ~~A total of 60~~Sixty male SD rats were randomly divided into four groups: control ~~group~~ (CG), nephritis ~~group~~ (NG), nephritis~~+~~ + 1.25(OH~~)$_2$D$_3$ group~~)$_2$D$_3$ (NVG), and nephritis~~+~~ + 1.25(OH~~)$_2$D$_3$+~~)$_2$D$_3$ + rapamycin ~~group~~ (NVRG) (n=15 per group~~), where3~~). Three rats from each group were ~~randomly killed~~sacrificed on ~~day~~days 1, 3, 7, 14, and 21 after ~~the~~ intervention.~~The urine~~ Urine samples were collected from the rats ~~for 24h~~over 24 hours on day 0 to ~~determine~~measure urinary protein excretion~~ at day 0.To quantify the pathological severity of renal injury, renal~~. Renal tissue samples were stained with HE and PAS~~, –~~ to assess the severity of renal injury, and immunohistochemistry was used to measure ~~the expression of~~ PCNA and mTOR expression in the renal tissues.

Results: Compared to NG, mesangial cell proliferation in the renal tissues was significantly alleviated in NVG and NVRG at all time points (all $p<0.05$); PCNA expression was significantly ~~increased~~higher in the NG compared to the CG ($p < 0.05$) and significantly ~~decreased~~lower in the NVG and NVRG ($p < 0.05$~~); however,.significantly increased in~~). mTOR ~~in~~ expression was also significantly increased in the NG compared ~~with~~to the CG, ~~and significantreduction in mTOR~~with a significant reduction observed in the NVG and NVRG compared ~~with~~to the NG.

Conclusion: Our ~~results~~ findings ~~revealed~~ clarify that 1.25(OH)$_2$D$_3$ could significantly inhibit the proliferation of glomerular mesangial cells in rats. Also, mTOR protein was involved in the process of 1.25(OH)$_2$D$_3$ regulating glomerular mesangial cells. Our ~~findings~~

Formatted: Font: 16 pt

Formatted: Font: 16 pt

Formatted: Font: 16 pt

results further elucidate the molecular mechanism of 1.25$(OH)_2D_3$ in alleviating renal injury in glomerulonephritis.

Formatted: Font: Not Bold, Not Italic

Formatted: Font: 16 pt

Keywords: 1.25$(OH)_2D_3$, mammalian target of rapamycin (mTOR), Thy-1 Nephritis, Proliferation

Introduction

Glomerulonephritis is ~~the~~a common cause of end-stage renal disease (ESRD~~)[1]. According to data~~) [1]. Data from the National Dialysis Patient Registry of the China Renal Data System~~,glomerulonephritides are~~ indicate that glomerulonephritis is the leading cause of ESRD in China~~[2, 3].~~ [2, 3]. The key pathological features of glomerulonephritis include the proliferation of mesangial cells and abnormal deposition of extracellular ~~matrices. Patients~~matrix. Early treatment of patients with nephrotic syndrome ~~can be treated with~~using steroids ~~early on,thusachieving~~can lead to better long-term remission ~~[4]. Nonetheless~~[4]. However, in patients with membranous glomerulopathy or proliferative nephritis, steroids alone have shown only modest improvement in renal parameters~~[5].~~ [5]. Therefore, exploring novel therapeutic agents for treating mesangioproliferative glomerulonephritis and preventing ESRD ~~prevention~~ is ~~of utmost importance~~critically important.

1,25-Dihydroxyvitamin ~~D3~~ D_3 (1,25(OH~~)2D3)~~ $)_2D_3$) is ~~an~~the active form of vitamin ~~D3, which~~ D_3 and has been reported to ~~have~~possess anti-inflammatory~~ and~~, anti-tumor, and cardiovascular-protective properties~~[6-8]. After proper~~ [6-8]. Proper treatment~~,~~ can induce apoptosis in proliferated mesangial cells~~ can clear themselves through apoptosis so the~~ , allowing for the recovery of glomerular structure~~ recovers~~, as ~~shown by~~demonstrated in the Thy-1 nephritis model~~[9]. The inhibited~~ [9]. Inhibiting the proliferation of mesangial cells can delay ~~the~~disease progression and improve ~~the~~ prognosis~~ of the disease.~~. Previous studies have shown that 1~~.~~,25(OH~~$)_2D_3$~~$)_2D_3$ can significantly inhibit the proliferation of mesangial cells cultured in ~~*vitro*and induce~~ vitro and promote their differentiation and apoptosis~~[10, 11].~~ [10, 11]. However, it remains unclear whether 1,25(OH~~)2D3~~$)_2D_3$ can regulate the pathology and progression of glomerulonephritis through mTOR~~ is yet to be determined.~~ .

~~Ithas~~mTOR has been reported ~~that mTOR is~~to be involved in mesangial cell proliferation and oxidative damage in the development of chronic kidney diseases, such as diabetic

Formatted: Font: Times New Roman

Formatted: Font: Not Italic

nephropathy and IgA nephropathy~~[12, 13]. Activating~~ [12, 13]. Activation of the phosphoinositide 3-kinase (PI3K)/protein kinase (Akt)/mammalian target of rapamycin (mTOR) ~~signal transduction~~signaling pathway can ~~affect~~influence cell proliferation, hypertrophy, and apoptosis~~[14]. Nonetheless~~ [14]. However, the role of the mTOR ~~signal transduction~~signaling pathway in the ~~process of $1.25(OH)_2D_3$ regulating~~regulation of mesangial cells by $1.25(OH)\ _2D_3$ has been rarely ~~reported.~~ explored.

In ~~the present~~this study, ~~weestablishedthe~~we established Thy-1 rat nephritis models~~, after which we gave~~ and administered 1.25(OH~~$)_2D_3$~~$)\ _2D_3$ and rapamycin ~~intervention~~ to ~~rats in~~ the ~~experiment~~experimental groups to observe ~~the~~ changes in PCNA and mTOR ~~expressions~~expression in rat renal tissues at different time points.We also ~~made~~conducted a preliminary ~~discussion about~~investigation into the ~~effect~~ target and mechanism of 1.25(OH~~$)_2D_3$~~$)\ _2D_3$ in regulating mesangial ~~cellsto~~cells, aiming to provide a reliable theoretical basis for further ~~studies~~research in ~~the~~this field.

Methods

Animals

~~All 60 clean-level~~Sixty male SD rats (6 weeks old~~ and~~, weighing 180~~ to 200g~~-200 g) were ~~purchased~~obtained from the Xinjiang Center for Local Epidemic Disease Control. ~~All the animals~~The rats were housed in an environment with a temperature of 22 ± 1 ºC, relative humidity of 50 ± 1%, and a 12/12-hour light/dark cycle~~ of 12/12 hr~~, with noise ~~level< 85dB, and~~ levels below 85 dB. They were ~~given~~ provided with food and water ~~and food~~ ad libitum.All animal ~~studies (~~procedures, including ~~the mice~~ euthanasia~~ procedure)~~, were approved by the Shihezi University ~~institutional animal care~~Institutional Animal Care and Use Committee and were ~~done~~conducted in ~~compliance~~accordance with the regulations and animal welfare guidelines of Shihezi University ~~institutional animal care~~ (SCXK ~~(~~[Xin~~)~~] 2013-178).~~Animals~~ The animals were randomly ~~grouped~~divided into four ~~following~~ groups (15 rats per group): control group (CG), ~~the~~ nephritis group (NG), ~~the~~ nephritis~~+~~ +

Formatted: Font: Times New Roman

Formatted: Font: Not Italic

1.25(OH)$_2$D$_3$group) $_2$D$_3$ group (NVG), and the nephritis+ + 1.25(OH)$_2$D$_3$+)$_2$D$_3$ + rapamycin group (NVRG).

Thy-1 nephritis induction and treatment

Formatted: Font: Times New Roman

Thy-1 nephritis models were prepared as described previously [15].described [15]. Briefly, the rats in the NG group, NVG group, and NVRG group were givengroups received a single tail vein injection of 25uL/100g25 μL/100 g of Thy-1 monoclonal antibodies (Ontario, Canada, catalog No.: CL005A), and thewhile rats in the CG were given a received an equivalent dose of saline injection of the same dose.. After the injection, urine was collectedfrom rats in fourcollected from all groups for 24hover 24 hours using metabolic cages, and proteinuria in fresh urine was examined byassessed using the standard Coomassie blue staining assay. After successful modeling, rats in the NVG received a gastric perfusion of 0.5ug5 μg/day of of 1.25(OH)$_2$D$_3$) $_2$D$_3$ (Shanghai Roche Pharmaceuticals Ltd.) dissolved in 1ml1 mL of peanut oil, and rats in the NVRG received a gastric perfusion of 30.5ug0.5 μg/day of 1.25(OH)$_2$D$_3$) $_2$D$_3$ and 1mg1 mg/day of rapamycin (Yixinke, Research Institute of North China Pharmaceutical Group) dissolved in 1ml1 mL of peanut oil. Rats in the CG and NG were given a gastric perfusion of 1ml1 mL/day of peanut oil. The rat model of Thy-1 nephritis model has a self-healing property, so we chose to observe the results of the experiment on dayobservations were made on days 1, 3, 7, 14, and 21. On day 1, 3, 7, 14, and 21after Thy-1 nephritis induction and drug treatment, 3 rats inthese days, 3 rats from each group were randomly sacrificed((using 2% pentobarbital sodium, 30 mg/kg, intraperitoneally), and kidney tissues were collected.

Formatted: Pattern: Clear

Hematoxylin and eosin (HE) staining and Periodic Acid-Schiff (PAS) staining

Formatted: Font: Times New Roman

For HE staining, renal tissues were fixed in 10% formaldehyde medium, embedded in

paraffin, and ~~cut~~sectioned into 3-~~μm~~μm-thick ~~sections. Tissue~~slices. The sections were baked at 60°C for ~~2h,~~2 hours, then placed in xylene and ethanol for dewaxing and hydration~~,~~. They were stained with hematoxylin for 5~~ min~~ minutes and eosin for 3~~ min~~ minutes. For PAS staining, ~~tissue~~ sections were ~~reacted~~treated with periodic acid~~. After being washed, they were incubated~~, followed by washing and incubation with Schiff's reagent, and then stained with hematoxylin. After dehydration with ethanol and xylene, the ~~tissue~~ sections were sealed with neutral gum.

~~According~~ Pathological damage was graded according to the ~~grading~~ criteria for human mesangial proliferative glomerulonephritis (MPGN~~), the pathological damage was graded~~) as follows: grade 0, normal; grade I, mild ~~*mesangial*proliferation~~mesangial proliferation; grade II, open capillary loops~~,~~ with mesangial width not exceeding the capillary diameter, ~~presenting~~showing segmental distribution, and moderate ~~*mesangial*proliferation~~mesangial proliferation; grade III, compressed capillary loops~~, and the~~ with mesangial width exceeding the capillary diameter, ~~presenting~~showing diffuse distribution, severe proliferation, and ~~capillary loops that are~~ severely compressed~~, in addition to~~ capillary loops, along with the presence of nodules and clumpy solid areas.

Formatted: Default Paragraph Font, Font: 10.5 pt, Pattern: Clear

Immunohistochemistry

Formatted: Font: Times New Roman, Not Italic

Formatted: Font: Times New Roman

Immunohistochemistry was ~~performed~~conducted using a universal two-step PV-9000 test kit (ZSGB-BIO, China~~), following~~) according to the manufacturer's instructions. PCNA antibodies (#2586, 1:50) and mTOR antibodies (#2983, 1:2000) were ~~purchased~~obtained from Cell Signaling Technology. Five fields of view at 400X magnification were randomly ~~chosen in~~selected from each ~~evaluated~~ sample to ~~evaluate~~assess the percentage of positive cells and staining intensity. ~~Percentage~~The percentage of positive cells was scored as follows: 0 points~~,~~ for no staining~~;~~, 1 point~~,~~ for<10% staining~~;~~, 2 points~~:~~ for 10%-50% staining~~;~~, and 3 points~~,~~ for>50% staining. Staining intensity was scored as follows: 0 ~~point,~~points for no staining~~;~~, 1

Formatted: Default Paragraph Font, Font: 10.5 pt

point~~,~~, for light brownish~~-~~yellow~~;~~, staining, 2 points~~,~~, for brownish~~-~~yellow~~;~~, staining, and 3 points~~,~~, for brown staining. The final score was ~~generated~~calculated by ~~assessing the score for~~ multiplying the percentage of positive cells ~~multiplied~~score by the staining intensity score: negative ~~samples~~ (-) with a final score of 0; weak positive ~~samples~~ (+) with a final score of 2-3~~,~~; moderate positive ~~samples~~ (++) with a final score of 4-5; and strong positive ~~samples~~ (+++) with a final score of 7-9.

Statistical analysis

All data were presented as ~~the~~ mean ± standard deviation and analyzed using SPSS software (version 13.0; SPSS, Inc.). A paired t-test was used for paired samples. A nonparametric test was ~~used~~employed to compare the ~~expressions~~expression levels of PCNA and mTOR ~~protein~~proteins at different time points and among different groups. A P-value of <0.05 ~~represented a~~was considered statistically significant ~~difference~~.

Results

1.25(OH)~~2D3~~$_2D_3$ reduces proteinuria levels of Thy-1 nephritis rats

To ~~determine~~evaluate whether 1.25(OH~~)2D3)~~)$_2D_3$ can ~~ameliorate~~alleviate nephritis, ~~we measured the~~ proteinuria levels ~~of the rats~~ were measured in the CG ~~group~~, NG ~~group~~, NVG ~~group~~, and NVRG ~~group~~groups on ~~day~~days 0, 1, 3, 7,14, and 21~~, respectively.~~. The amount of water intake and ~~the~~urine volume ~~of urine produced in~~ across the four ~~groupsmeasured~~groups, as measured in metabolic cages, showed no ~~statistical significance~~statistically significant differences (p~~>~~>0.05). ~~The changes~~Changes in ~~the~~ urine protein ~~level~~levels are summarized in Table 1. Compared with the CG, ~~rats in~~the NG, NVG, and NVRG groups showed significantly higher ~~levels of~~ proteinuria levels on day 1, ~~indicating that~~ confirming the successful establishment of the Thy-1 nephritis rat model ~~was successfully established.~~. On ~~day~~days 3, 7,

Formatted: Font: Times New Roman

Formatted: Font: Not Italic

Formatted: Font: Times New Roman

Formatted: Font: Times New Roman

Formatted: Default Paragraph Font, Font: 10.5 pt

Formatted: Default Paragraph Font, Font: 10.5 pt

Formatted: Default Paragraph Font, Font: 10.5 pt

and 14, significant reductions in proteinuria were observed in ~~proteinuria of rats in~~the NVG and NVRG groups compared with ~~proteinuria of rats in~~the NG~~ (Figure 1, Table 1)~~, indicating that 1.25(OH)~~)2D3)~~ $_2$D$_3$ and its combination with rapamycin had a significant ~~alleviating~~ameliorating effect on Thy-1-induced nephritis~~.~~ (Figure 1, Table 1).

Table 1. Effect of 1.25(OH)$_2$D$_3$ treatment on proteinuria at different time points after the induction of glomerulonephritis

	day 0	Day 1	Day 3	Day 7	Day 14	Day 21
CG	1.517±0.712	1.119±0.434	1.528±0.107	2.748±0.569	1.769±0.174	2.54±0.439
NG	1.139±0.286	4.896±0.539*	10.98±0.156	8.04±1.699	4.45±0.354	2.482±0.594
NVG	0.786±0.181	3.015±0.397*	3.865±0.74#	5.53±1.057#	2.11±0.128#	2.241±0.688
NVRG	1.056±0.674	3.283±0.205*	3.157±0.205#	4.511±0.781#	1.808±0.593#	1.463±0.879

Note: $^{*}p<0.05$, compared to CG, indicates significant higher proteinuria on Day 1 after antibody injection. $^{*}p<0.05$, compared to CG, indicates significantly decreased mean urinary protein level of NVG on Day 3, Day 7, and Day 14. Unit: mg/d

1.25(OH)~~2D3~~ $_2$D$_3$ relieves renal injury in the Thy-1 induced nephritis rat model

The effect of 1.25(OH)~~)2D3)~~ $_2$D$_3$ on renal pathology was further ~~examined by~~ evaluated using HE and PAS staining (~~Figure~~Figures 2-3~~,).~~). The renal tissues of the CG ~~were~~appeared normal~~ and showed~~, with no proliferation of mesangial cells or ~~matrices, thus being graded~~extracellular matrix, resulting in a grade 0 for pathological damage. ~~By~~In contrast, the NG showed significant mesangial cell proliferation~~ of mesangial cells~~, severe ~~compressed~~ compression of capillary loops~~ and~~, nodules, and clumpy solid areas ~~were observed in NG~~ at all time points, ~~which aggravated from day 3 to day 7. Also, the~~ with the most severe pathological ~~renal~~ injury ~~was most severe~~ observed on day 7~~ and alleviated on~~, which then showed some alleviation by day 21. ~~Besides~~Additionally, some rats in the NG ~~even~~

~~had~~exhibited glomerular lobulation, sclerosis, and fibrosis~~ of the glomerulus.~~. Compared to the NG, mesangial cell proliferation in the ~~renal tissues~~NVG and NVRG groups was significantly ~~alleviated in NVG and NVRG~~ reduced at all time points (all $p<0.05$), ~~especially at day7~~particularly on days 7 and ~~day14, and the~~14. The capillary loops in these groups were only mildly compressed, with the mesangial ~~breadth~~width not ~~surpassing~~exceeding the capillary diameter and showing a segmental distribution (Table 2). These ~~results~~findings suggest that 1.25(OH~~)2D3 attenuates~~)$_2$D$_3$ reduces renal injury, including mesangial cell proliferation.

Formatted: Font: Not Bold

Table 2. Comparison of pathological damage grading of renal tissues among different groups at all time points

	Day 1	Day 3	Day 7	Day 14	Day21
CG	0.00±0.000	0.00±0.000	0.00±0.000	0.00±0.000	0.00±0.000
NG	0.67±0.516	1.33±0.516*	2.83±0.408*	2.00±0.632	0.83±0.754
NVG	0.83±0.408	1.00±0.632*	1.83±0.752#	1.17±0.408#	0.67±0.516
NVRG	0.67±0.516	0.83±0.753*	1.83±0.408#	1.00±0.632#	0.67±0.516

Note: $^*p<0.05$, indicates the severe renal pathological damage on compared to CG from Day 3. $^\#p<0.05$, indicates significantly alleviated pathological renal damage of NVG and NVRG on Day 7 and Day 14 compared to NG.

PCNA and mTOR expressions measured by immunohistochemistry

Formatted: Font: Times New Roman

~~Results~~The results above ~~found~~indicate that 1.25(OH~~)2D3~~)$_2$D$_3$ can suppress proteinuria and ~~alleviate~~reduce mesangial cell proliferation ~~of mesangial cells~~ and abnormal ~~deposition of~~ extracellular ~~matrices, indicating the~~ matrix deposition, demonstrating its protective ~~roles~~

~~of 1.25(OH)2D3~~effects on renal ~~functions~~function. To ~~further explore~~investigate the underlying mechanism, we ~~conducted~~performed immunohistochemistry to ~~evaluate the expression of~~ assess PCNA and mTOR~~. The positive~~ expression. PCNA expression ~~of PCNA~~ in mesangial cells ~~is presented~~appeared as brown-yellow granules located in the nucleus~~ or~~, occasionally observed in glomerular mesangial cells of normal rats~~ occasionally.~~. The quantification of PCNA ~~expressions~~expression is summarized in Table 3. Compared with the CG, the NG showed a significantly increased level of PCNA expression ~~level~~ in renal tissues (p<0.05) and in glomerular mesangial cells on ~~day~~days 1, 3, 7, 14, and 21~~, and the~~. The PCNA expression ~~level of PCNA~~ in the NG increased over time~~ (from day 1 to~~, peaking on day 7~~), consistent~~, which aligns with the trend in ~~the~~ mesangial cell proliferation~~ score~~. Compared ~~with~~to the NG, both the NVG and NVRG ~~showed a~~ groups exhibited significantly ~~decreased~~lower PCNA expression in glomerular mesangial cells ~~than NG~~ at all time points (p<0.05~~) and had~~), with a ~~lower~~reduction in PCNA expression observed on ~~both day~~days 14 and ~~day~~ 21 ~~than on~~compared to day 7 (Figure 4) (p<0.05).

~~The positive~~mTOR expression ~~of mTOR~~ in mesangial cells ~~is presented~~was visualized as nest-like, cord-like, or diffusely distributed brown-yellow granules located in the nucleus. The expression levels of mTOR ~~expressions of~~across different groups ~~at different~~and time points are summarized in Table 4. In the NG~~ group, we found that~~, mTOR expression ~~level~~ was significantly ~~increased~~higher in renal tissues and glomerular mesangial cells compared to the CG (p<0.05~~) compared with CG. At~~). In the ~~same time~~CG, there were no significant ~~intragroup differences at all~~changes in mTOR expression across the different time points (all p>0.05~~)~~). However, in the ~~CG group. Also, the~~NVG and NVRG groups, mTOR expression ~~level~~ was significantly ~~decreased in NVG and NVRG~~lower compared ~~with~~to the NG ~~group~~ at all time points (p<0.05),~~while the~~ with mTOR expression ~~decreased~~levels decreasing over time (Figure 5).

Formatted: Font: Not Bold

Formatted: Font: Not Bold

Formatted: Font: Not Bold

Formatted: Font: Not Bold

Table 3. Comparison of PCNA expression grading among different groups at different time points

	day 1	day 3	day 7	day 14	day 21
CG	0.00±0.00●	0.00±0.00	0.00±0.00▲	0.00±0.00	0.00±0.00
NG	3.41±1.23●	5.44±2.09	8.14±2.65▲	6.58±1.86	2.92±1.51
NVG■	3.62±2.97	4.33±0.67	4.81±1.16★	4.42±1.56	2.47±1.17
NVRG■	2.57±1.07	3.53±1.39	5.27±2.18※	4.75±2.17	2.11±1.63

Note: indicates PCNA expression in renal tissues on Day 1 after antibody injection and ●p<0.01; indicates the highest PCNA expression in renal tissues on Day 7 after antibody injection and ▲p<0.01; indicates a lower PCNA expression of NVG than that of NG on Day 7 and ★p<0.05; ※ indicates a decreased PCNA expression of NVRG on Day 7 and p<0.05; indicates no significant difference in PCNA expression between NVG and NVRG at all time points and ■p>0.05.

Table 4. Comparison of mTOR expression grading among different groups at different time points

	day 1	day 3	day 7	day 14	day 21
CG	1.33±0.58●	2.00±2.00	1.33±2.31▲	2.00±2.00	2.33±1.52
NG	6.00±3.00●	8.00±1.00	12.00±0.00▲	8.00±1.00	3.33±1.15
NVG■	4.67±1.15	4.67±1.15	10.00±1.00★	3.33±1.15	2.33±1.15
NVRG■	3.81±1.15	3.91±2.31	10.00±3.00※	4.46±1.15	3.00±2.00

Note: indicates mTOR expression in renal tissues on Day 1 after antibody injection and ●p<0.01; indicates the highest mTOR expression in renal tissues on Day 7 after antibody injection and ▲p<0.01; indicates a lower mTOR expression of NVG than that of NG on Day 7 and ★p<0.05; ※ indicates a decreased mTOR expression of NVRG on Day 7 and p<0.05; indicates no significant difference in mTOR expression between NVG and NVRG at all time points and ■ p>0.05.

The expression of PCNA expression and mTOR is associated with

Formatted: Font: Times New Roman

a degree of renal pathological in Thy-1 nephritis rats

The ~~association~~relationship between PCNA or mTOR expression and renal pathological damage was analyzed, revealing that the ~~quantitative 24h~~24-hour urinary protein ~~result~~levels, PCNA expression, and mTOR expression were positively correlated with each other ($p<0.05$), and all~~ of them~~ were positively correlated with the pathological damage grading ($p<0.05$), as shown in Table 5.

Table 5. Correlation among pathological damage grading, PCNA expression, and mTOR expression in glomerular renal tissues

	r	P
Pathological damage and mTOR	0.729	P<0.001
Pathological damage and PCNA	0.652	P<0.001
PCNA and mTOR	0.677	P<0.001

Discussion

Abnormal proliferation of mesangial cells, which are key intrinsic cells of the glomerulus, ~~and~~along with the release of inflammatory mediators and pathological accumulation of extracellular matrices, are ~~the essential links~~critical factors leading to glomerular sclerosis and the ~~development~~progression of ~~different~~various glomerulonephritides to end-stage renal ~~diseases[16, 17].~~disease [16, 17]. Therefore, ~~exploring~~developing new drugs and ~~therapy~~therapeutic strategies for glomerulonephritis ~~by maintaining the~~ that maintain a balance between mesangial cell proliferation, hypertrophy, and apoptosis~~ of mesangial cells and~~, while improving the metabolism of mesangial matrices, is essential to delay or reverse glomerular sclerosis. Previous studies have shown that 1.25(OH~~$)_2D_3$~~$)\ _2D_3$ can inhibit ~~the~~ mesangial cell proliferation ~~of mesangial cells~~ and induce apoptosis ~~of mesangial cells~~ by blocking the cell cycle~~[18, 19];~~ [18, 19]; however, the underlying mechanism ~~remained~~remains unclear. ~~Herein~~In this study, we ~~constructed~~established a nephritis rat model induced by Thy-1 injection and ~~assessed the~~evaluated renal ~~pathologies~~pathology to determine the roles and underlying mechanisms

Formatted: Font: Not Bold

of 1.25(OH)$_2$D$_3$ on) $_2$D$_3$ in glomerulonephritis.

Compared with the CG, the experimental groups developed significant proteinuria on day 1 after antibody injection that, which peaked on day 3 and returned to the normal level onlevels by day 14, possiblylikely due to the self-healing tendencynature of the Thy-1 nephritis model. The NVG had theshowed lower urinary protein level lowerlevels than the NG at all time points, suggestingindicating that 1.25(OH)$_2$D$_3$) $_2$D$_3$ could lowerreduce proteinuria and protect the urinary protein level, thus protecting the kidney, which was in linekidneys, consistent with the results reported byfindings of Szeto CC[14]. Moreover, compared with NG, [14]. Additionally, the NVG hadexhibited significantly milder histomorphologichistopathological changes compared to the NG at all time points, such as significantlyincluding reduced mesangial cells andcell proliferation, decreased extracellular matricesmatrix accumulation, alleviated accumulation of inflammatory factorsfactor buildup, and scatteredfewer balloon adhesions observed on day 7 in pathological test results of the kidney, thus suggesting that . These findings suggest that 1.25(OH)2D3 regulates) $_2$D$_3$ mitigates Thy-1 nephritis not only by suppressing the urinary protein levelreducing proteinuria but also by causing histomorphologicinducing favorable histopathological changes.

mTOR participatesplays a key role in cell survival, proliferation, and apoptosis[20]. [20]. It is usuallyoften continuously activated in many tumor cells, suggesting that it is closely related to the indicating its close relationship with the proliferation, survival, and metastasis of tumor cells [21].these cells [21]. A recent study showeddemonstrated that mTOR is up-regulatedupregulated in mice with lupus nephritis and is strongly related toassociated with the pathological proliferation of mesangial cells[10]. [10]. 1.25(OH)$_2$D$_3$) $_2$D$_3$ has been provenshown to induce differentiation and inhibit thecell proliferation of cells in differentvarious tissues. NeverthelessHowever, whether mTOR mediates itsthis biological

effect and whether there is a correlation between them remains ~~unknown~~unclear. In ~~their~~a 2006 study~~ conducted in 2006~~, Regulska et al. found that mTOR ~~was~~is involved in the protection of nerve cells by 1.25(OH~~$)_2D_3$~~) $_2D_3$ and its analogs~~[22] and was also involved in the process of 1.25(OH)$_2D_3$ and its receptors inhibiting~~ [22], as well as in the inhibition of staurosporine-mediated apoptosis of osteoblasts~~[23].~~ by 1.25(OH) $_2D_3$ and its receptors [23]. Currently, there are ~~only a~~ few reports on the ~~correlation~~relationship between mTOR and ~~the~~ mesangial cell proliferation~~ of mesangial cells.~~. In ~~the present~~this study, mTOR expression in the experimental groups ~~had a mTOR expression on day 1, which~~ increased ~~on~~from day ~~3~~1, peaked on day 7, and then gradually declined. Both NVG and NVRG had ~~a~~ lower mTOR expression ~~than~~ levels compared to NG at all time points ($p<0.05$), but ~~the difference in mTOR expression between NVG and NVRG was not~~ there was no statistically significant difference between NVG and NVRG ($p>0.05$). The expression of mTOR was positively correlated with the expression of PCNA, a cell cycle regulatory protein, suggesting that 1.25(OH~~$)_2D_3$ could~~) $_2D_3$ may inhibit mTOR expression~~,~~, and, thus~~ inhibiting,~~ the proliferation of mesangial cells. We also ~~found~~observed that the combined intervention of rapamycin and 1.25(OH~~$)_2D_3$~~) $_2D_3$ did not ~~cause~~produce a ~~superimposed~~synergistic effect in rats with nephritis. There was ~~also~~ no significant difference in mTOR expression between ~~such~~ rats~~,~~ treated with both agents and ~~rats were intervened only~~those treated with 1.25(OH~~$)_2D_3$, so a synergistic effect of 1.25(OH)$_2D_3$ and~~) $_2D_3$ alone, suggesting that rapamycin ~~was not proven. We speculate that rapamycin~~ and 1.25(OH~~$)_2D_3$~~) $_2D_3$ may share the same ~~effect~~ target~~:~~, the mTOR protein. ~~As for the~~The specific mechanism ~~of~~by which 1.25(OH~~$)_2D_3$ inhibiting~~) $_2D_3$ inhibits mTOR expression~~, the issue~~ should be ~~addressed~~explored further from ~~the perspective of~~a molecular biology perspective.

~~We comprehensively analyzed~~Our comprehensive analysis of the effects of 1.25(OH~~$)_2D_3$~~) $_2D_3$ on renal histopathology, mTOR protein, and PCNA in rat nephritis models~~, showing~~

Formatted: Font: Not Italic

showed that the grading of pathological renal damage, PCNA expression, and mTOR expression were positively correlated ~~with each other~~ (p<0.05). ~~This further suggested~~These findings suggest that mTOR and PCNA ~~could~~may be involved in the ~~1.25(OH)2D3 inhibiting rat~~inhibition of mesangial cell proliferation and ~~alleviating~~the alleviation of renal injury~~.~~ by 1.25(OH)$_2$D$_3$.

The present study has some limitations, as we did not ~~verify~~explore the possible molecular mechanisms ~~and conducted more in~~ in greater depth~~ studies~~. In our future ~~studies~~research, we will ~~perform~~conduct PCNA and mTOR knockdown experiments to further validate ~~furthertheir~~their roles in the protective ~~effect~~effects of 1.25(OH~~)2D3~~)$_2$D$_3$ against ~~the development of~~ glomerulonephritis.~~As another limitation that~~Additionally, only a single dose of 1.25(OH~~) 2D3~~)$_2$D$_3$ was used~~, and that~~ in this study, so different doses will be investigated in future studies.

Formatted: Indent: First line: 0.29"

Formatted

Formatted

Formatted

In conclusion, our results ~~suggested~~suggest that 1.25(OH~~)$_2$D$_3$~~)$_2$D$_3$ significantly ~~inhibited the~~inhibits mesangial cell proliferation ~~of mesangial cells~~ in rats, ~~which may be exerted~~potentially through ~~decreasing~~the reduction of mTOR and PCNA expression in nephrotic rats. ~~Also, the~~The mTOR protein may be a key target of the biological ~~effect~~effects of 1.25(OH~~)$_2$D$_3$ might be the mTOR protein.~~)$_2$D$_3$.

Acknowledgments

Formatted: Font: 16 pt

Not applicable.

Funding

Formatted: Font: 16 pt

This work was supported by the National Natural Resources Foundation of China (81160090) and the Research Project Supported by Xinjiang Production and Construction Corps of China (2009GG59). The funders had no role in study design, data collection and analysis, decision to publish, or preparation of the manuscript.

Formatted: Space Before: 0 pt, After: 0 pt, Line spacing: Double

Availability of data and materials

Formatted: Font: 16 pt

All data generated or analyzed during this study are included in this article.

Authors' contributions

Formatted: Font: 16 pt

All authors contributed to the ~~study~~study's conception and design. Xiao-ping Yang conceived and supervised the study; Xiao-ping Yang and Jian-feng Li designed the experiments; Jian-feng Li and Jie Suo performed the experiments; Lei Jin and Huan Ma analysed the data; Jian-feng Li and Lei Jin wrote the manuscript; Xiaoping Yang and Rui Yang ~~made~~revised the manuscript ~~revisions~~. All authors reviewed the results and approved the final version of the manuscript.

Consent to Participate

Formatted: Font: 16 pt

Not applicable.

Consent to Publish

Formatted: Font: 16 pt

Not applicable.

Competing interests

Formatted: Font: 16 pt

The authors have no relevant financial or non-financial interests to disclose.

Financial interests~~:~~

Formatted: Font: 16 pt

The authors declare they have no financial interests.

References

Formatted: Font: 18 pt

1. Wang F, Yang C, Long J, et al. Executive summary for the 2015 Annual Data Report of the China Kidney Disease Network (CK-NET). Kidney international. 2019;95(3): 501-505. PMID: 30784660

2. Wei X, Li Z, Chen W, et al. Prevalence and risk factors of chronic kidney disease in first-degree relatives of chronic kidney disease patients in Southern China. Nephrology (Carlton, Vic).2012; 17(2): 123-130. PMID: 21919998

3. Liu ZH. Nephrology in china. Nature reviews Nephrol. 2013;9(9): 523-528. PMID: 23877587

4. Lieberman KV ,Pavlova-Wolf A. Adrenocorticotropic hormone therapy for the treatment of idiopathic nephrotic syndrome in children and young adults: a systematic review of early clinical studies with contemporary relevance. Journal of nephrology. 2017;30(1): 35-44. PMID: 27084801

5. Heidy Hendra ,Alan D Salama. Steroids as treatment for glomerulonephritis: time for a rethink. Nephrology, dialysis, transplantation : official publication of the European Dialysis and Transplant Association - European Renal Association . 2022;37(7): 1212-1217. PMID: 33188694

6. Ayse Sagmak Tartar , Safak Ozer Balin .Geriatric urinary tract infections: The value of laboratory parameters in estimating the need for bacteremia and Intensive Care Unit. Pakistan journal of medical sciences. 2019;35(1): 215-219. PMID: 30881426

7. Han J, Tang Y, Zhong M and Wu W.Antitumor effects and mechanisms of 1,25(OH)2D3 in the Pfeiffer diffuse large B lymphoma cell line. Molecular medicine reports. 2019;20(6): 5064-5074. PMID: 31638226

8. Da J, Yang Y, Dong R, Shen Y and Zha Y. Therapeutic effect of 1,25(OH)2-VitaminD3 on fibrosis and angiogenesis of peritoneum induced by chlorhexidine. Biomedicine & pharmacotherapy. 2020;129: 110431. PMID: 32585450

9. Hughes J, Savill JS. Apoptosis in glomerulonephritis. Current opinion in nephrology and hypertension. 2004;30(3): 655-676. PMID: 15261347

10. Abe H, Iehara N, Utsunomiya K, Kita T and Doi T.A vitamin D analog regulates mesangial cell smooth muscle phenotypes in a transforming growth factor-beta type II receptor-mediated manner. The Journal of biological chemistry. 1999;274(30): 20874-20878. PMID: 10409630

11. Weinreich T, Merke J, Schönermark M, et al. Actions of 1,25-dihydroxyvitamin D3 on human mesangial cells. American journal of kidney diseases. 1991;18(3): 359-366. PMID: 1652888

12. Han F, Xue M, Chang Y, et al. Triptolide Suppresses Glomerular Mesangial Cell Proliferation in Diabetic Nephropathy Is Associated with Inhibition of PDK1/Akt/mTOR Pathway. International journal of biological sciences. 2017;13(10): 1266-1275. PMID: 29104493

13. Xia M, Liu D, Tang X, et al. Dihydroartemisinin inhibits the proliferation of IgAN mesangial cells through the mTOR signaling pathway. International immunopharmacology. 2020;80: 106125. PMID: 31931362

14. LoPiccolo J, Blumenthal GM, Bernstein WB and Dennis PA. Targeting the PI3K/Akt/mTOR pathway: effective combinations and clinical considerations. Drug resistance updates : reviews and commentaries in antimicrobial and anticancer chemotherapy. 2008;11(1-2): 32-50. PMID: 18166498

15. Hashimoto K, Kamijo Y, Nakajima T, et al. PPARα Activation Protects against Anti-Thy1 Nephritis by Suppressing Glomerular NF-κB Signaling. PPAR research . 2012;2012: 976089. PMID: 22675338

16. Lei D, Chengcheng L, Xuan Q, et al. Quercetin inhibited mesangial cell proliferation of early diabetic nephropathy through the Hippo pathway. Pharmacological research. 2019;146: 104320. PMID: 31220559

17. Liu L, Ge W, Zhang Z, et al. Sublytic C5b-9 triggers glomerular mesangial cell proliferation via enhancing FGF1 and PDGFα gene transcription mediated by GCN5-dependent SOX9 acetylation in rat Thy-1 nephritis. FASEB journal : official publication of the Federation of American Societies for Experimental Biology. 2021;35(7): e21751. PMID: 34156114

18. Zhang CJ, Zhao D, Yin X, et al. Effects of 1,25(OH)2D3 on proliferation and apoptosis of human glomerular mesangial cells. American journal of translational research. 2016;8(6): 2659-2666. PMID: 27398149

19. Panichi V, Migliori M, Taccola D, et al. Effects of 1,25(OH)2D3 in experimental mesangial proliferative nephritis in rats. Kidney international. 2001;60(1): 87-95. PMID: 11422740

20. Suto T and Karonitsch T. The immunobiology of mTOR in autoimmunity. Journal of autoimmunity. 2020;110: 102373. PMID: 31831256

21. Murugan AK. mTOR: Role in cancer, metastasis and drug resistance. Seminars in cancer biology. 2019; 59: 92-111. PMID: 31408724

22. Zhang C, Chan CCY, Cheung KF, et al. Effect of mycophenolate and rapamycin on renal fibrosis in lupus nephritis. Clin Sci (Lond). 2019;133(15):1721-1744. PMID: 31358596

23.Regulska M, Leśkiewicz M, Budziszewska B, et al. Inhibitory effects of 1,25-dihydroxyvitamin D3 and its low-calcemic analogues on staurosporine-induced apoptosis. Pharmacological reports : PR. 2007;59(4): 393-401. PMID: 17901567

24. Zhang X and Zanello LP. Vitamin D receptor-dependent 1 alpha,25(OH)2 vitamin D3-induced anti-apoptotic PI3K/AKT signaling in osteoblasts. Journal of bone and mineral research : the official journal of the American Society for Bone and Mineral Research. 2008; 23: 1238-1248. PMID: 18410228

Figure legends

Figure 1 Measurement of proteinuria levels in CG, NG, NVG, and NVRG.

Figure 2HE staining images of renal tissues (×400).

Figure 3PAS staining images of pathological changes in renal tissues among 4 groups (×400).

Figure 4 Representative immunohistochemistry images of PCNA expression in renal tissues among CG, NG, NVG, and NVRG (×400).

Figure5 Representative immunohistochemistry images of mTOR expression in renal tissues among CG, NG, NVG, NVRG (×400).

Dear Editor,

Thank you for carefully reviewing our manuscript previously titled“**1.25(OH)2D3 decrease PCNA and mTOR expression and alleviates renal injury in Thy-1 nephritis rat model**” for possible publication in the **PLOS One** . Weare grateful to you and your reviewers for their constructive critique. We have revised the manuscript, highlighting our revisions in red. and have attached point-by-point responses detailing how we have revised the manuscript in response to the reviewers' comments below.

Reviewer 1

1. In the Abstract you mention "thylakoid cells" in the Objective. Should this have been "glomerular mesangial cells"?

Response:

First of all, thank you very much for your patience and careful review. In fact, our team spent a long time in translating this manuscript between the words "mesangial" and "thylakoid", and finally chose "thylakoid", which we meant to express glomerular mesangial cells. So, following your instructions, we changed "thylakoid" to "mesangial" in our manuscript. Thank you again for your review.

2. I would have loved to see Tables III and IV in graphs, but understand if there wasn't enough room.

Response:

I'm sorry. When we submitted our manuscript to PLOS One, we uploaded the manuscript, pictures and tables separately, so you didn' t see all the tables. According to PLOS One's requirements, we have included tables as part of main manuscript and remove the individual files. Please review them again.

3. In Table V, you don't need the lower left triangular region, which is simply a reflection of the upper right triangular region.

Response:

In order to improve the data presentation effect, our research team discussed to modify Table V based on your comments. We have put the new Table V in the revised manuscript. Please review it again.

4. Do you care to speculate on the relative efficacy of Vitamin D vs rapamycin? Has rapamycin already been shown to be effective by itself? For example, does this paper show that? Lock, Helen R., Steven H. Sacks, and Michael G. Robson. "Rapamycin at subimmunosuppressive levels inhibits mesangial cell proliferation and extracellular matrix production." American Journal of Physiology-Renal Physiology 292.1 (2007): F76-F81.

Response:

First of all, thank you very much for your reference, we have studied it carefully. This study, like many international studies, has demonstrated that low doses of rapamycin can significantly inhibit mesangial cell proliferation and mesangial cell matrix deposition. Second, the inhibitory effect of rapamycin on mesangial cells is directed towards the mTOR protein, which has been confirmed by a large number of previous studies (1).Third, our research group has been concerned about the biological activity of 1.25(OH)2D3 for a long time and has carried out a lot of research. We observed that 1.25(OH)2D3 inhibits rat mesangial cells cultured in vitro, and this was also observed in rat animal experiments(2). Fourth, some studies have shown that 1.25(OH)2D3 inhibits tumor cell proliferation by inhibiting mTOR signaling pathway(3).Based on these findings, we venture to speculate whether the inhibition of mesangial cell proliferation by 1.25(OH)2D3 also affects the mTOR signaling pathway. In this study, there was no significant difference in mTOR expression between NVG and NVRG groups， Rapamycin and 1.25(OH)2D3 had no additive effect on mTOR protein inhibition in NVRG group. As rapamycin can significantly inhibit mTOR protein, we hypothesized that rapamycin and 1.25(OH)2D3 may have an intersection in regulating mesangial cell proliferation or may inhibit the same cell signaling pathway. However, further molecular biology research is needed to explore the pathway by which 1.25(OH)2D3 inhibits mTOR

protein. Therefore, in this study, we concluded that mTOR protein was involved in the process of 1.25 (OH) 2D3 regulating glomerular mesangial cells.

(1) Bashier Osman 1, El-Sayed Akool. Differential modulation of the cytokine-induced MMP-9/TIMP-1 protease-antiprotease system by the mTOR inhibitor rapamycin. Biochem Pharmacol;. 2011 Jan 1;81(1):134-43.PMID: 20854798

(2) Gang Liu , Guochen Ren , Xiaoping Yang .1, 25(OH)2D3 regulates the proliferation, fibrosis, and autophagy of mesangial cells induced by high glucose via the VDR/mTOR pathway. Xi Bao Yu Fen Zi Mian Yi Xue Za Zhi. 2022 Mar;38(3):224-230. PMID: 35365987

(3) Jing Yang , Takayuki Ikezoe, Chie Nishioka .Inhibition of mTORC1 by RAD001 (everolimus) potentiates the effects of 1,25-dihydroxyvitamin D(3) to induce growth arrest and differentiation of AML cells in vitro and in vivo. Exp Hematol. 2010 Aug;38(8):666-76. PMID: 20382200

Reviewer 2: This is an interesting piece of work. The findings provide more insight on the role of 1.25(OH)2D3 on glomerulonephritis. The authors should check that the article is clear and concise. Grammatical errors were present in the abstract and manuscript.

Response:

Thank you for your recognition and encouragement. We deeply evaluate and revise the language, grammar, and logic in the manuscript and invite professional scientific editing service (MedSci, Shanghai Metz Pharmaceutical Technology Co., Ltd) to help us，which meets PLOS One's requirements.

Thank you for your consideration and further review of our manuscript. Please do not hesitate to contact us with any further questions or recommendations.

Yours Sincerely,

Xiaoping Yang

Jianfeng Li

Lei Jin
