# Supplementary material for: 1.25(OH)2D3 decreases PCNA and mTOR expression and alleviates renal injury in Thy-1 nephritis rat model
Source: PLoS One. 2024 Dec 5;19(12):e0311000. doi: 10.1371/journal.pone.0311000 (PMC11620346; doi:10.1371/journal.pone.0311000)
Supplement: Attachment — Submitted filename: Response to Reviewers.docx [file pone.0311000.s001.docx]

Dear Editor,

Thank you for carefully reviewing our manuscript previously titled“**1.25(OH)2D3 decrease PCNA and mTOR expression and alleviates renal injury in Thy-1 nephritis rat model**” for possible publication in the **PLOS One** . Weare grateful to you and your reviewers for their constructive critique. We have revised the manuscript, highlighting our revisions in red. and have attached point-by-point responses detailing how we have revised the manuscript in response to the reviewers' comments below.

**Reviewer 1**

**1. In the Abstract you mention "thylakoid cells" in the Objective. Should this have been "glomerular mesangial cells"?**

**Response:**

First of all, thank you very much for your patience and careful review. In fact, our team spent a long time in translating this manuscript between the words "mesangial" and "thylakoid", and finally chose "thylakoid", which we meant to express glomerular mesangial cells. So, following your instructions, we changed "thylakoid" to "mesangial" in our manuscript. Thank you again for your review.

**2. I would have loved to see Tables III and IV in graphs, but understand if there wasn't enough room.**

**Response:**

I'm sorry. When we submitted our manuscript to PLOS One, we uploaded the manuscript, pictures and tables separately, so you didn' t see all the tables. According to PLOS One's requirements, we have included tables as part of main manuscript and remove the individual files. Please review them again.

**3. In Table V, you don't need the lower left triangular region, which is simply a reflection of the upper right triangular region.**

**Response:**

In order to improve the data presentation effect, our research team discussed to modify Table V based on your comments. We have put the new Table V in the revised manuscript. Please review it again.

**4. Do you care to speculate on the relative efficacy of Vitamin D vs rapamycin? Has rapamycin already been shown to be effective by itself? For example, does this paper show that? Lock, Helen R., Steven H. Sacks, and Michael G. Robson. "Rapamycin at subimmunosuppressive levels inhibits mesangial cell proliferation and extracellular matrix production." American Journal of Physiology-Renal Physiology 292.1 (2007): F76-F81.**

**Response:**

First of all, thank you very much for your reference, we have studied it carefully. This study, like many international studies, has demonstrated that low doses of rapamycin can significantly inhibit mesangial cell proliferation and mesangial cell matrix deposition. Second, the inhibitory effect of rapamycin on mesangial cells is directed towards the mTOR protein, which has been confirmed by a large number of previous studies (1).Third, our research group has been concerned about the biological activity of 1.25(OH)2D3 for a long time and has carried out a lot of research. We observed that 1.25(OH)2D3 inhibits rat mesangial cells cultured in vitro, and this was also observed in rat animal experiments(2). Fourth, some studies have shown that 1.25(OH)2D3 inhibits tumor cell proliferation by inhibiting mTOR signaling pathway(3).Based on these findings, we venture to speculate whether the inhibition of mesangial cell proliferation by 1.25(OH)2D3 also affects the mTOR signaling pathway. In this study, there was no significant difference in mTOR expression between NVG and NVRG groups，Rapamycin and 1.25(OH)2D3 had no additive effect on mTOR protein inhibition in NVRG group. As rapamycin can significantly inhibit mTOR protein, we hypothesized that rapamycin and 1.25(OH)2D3 may have an intersection in regulating mesangial cell proliferation or may inhibit the same cell signaling pathway. However, further molecular biology research is needed to explore the pathway by which 1.25(OH)2D3 inhibits mTOR protein. Therefore, in this study, we concluded that mTOR protein was involved in the process of 1.25 (OH) 2D3 regulating glomerular mesangial cells.

(1) [Bashier Osman](https://pubmed.ncbi.nlm.nih.gov/?term=Osman+B&cauthor_id=20854798) [1](https://pubmed.ncbi.nlm.nih.gov/20854798/#full-view-affiliation-1), [El-Sayed Akool](https://pubmed.ncbi.nlm.nih.gov/?term=Akool+el-S&cauthor_id=20854798). Differential modulation of the cytokine-induced MMP-9/TIMP-1 protease-antiprotease system by the mTOR inhibitor rapamycin. Biochem Pharmacol;. 2011 Jan 1;81(1):134-43.PMID: 20854798

(2) [Gang Liu](https://pubmed.ncbi.nlm.nih.gov/?term=Liu+G&cauthor_id=35365987) , [Guochen Ren](https://pubmed.ncbi.nlm.nih.gov/?term=Ren+G&cauthor_id=35365987) , [Xiaoping Yang](https://pubmed.ncbi.nlm.nih.gov/?term=Yang+X&cauthor_id=35365987) .1, 25(OH)2D3 regulates the proliferation, fibrosis, and autophagy of mesangial cells induced by high glucose via the VDR/mTOR pathway. Xi Bao Yu Fen Zi Mian Yi Xue Za Zhi.  2022 Mar;38(3):224-230. PMID: 35365987

(3) [Jing Yang](https://pubmed.ncbi.nlm.nih.gov/?term=Yang+J&cauthor_id=20382200) , [Takayuki Ikezoe](https://pubmed.ncbi.nlm.nih.gov/?term=Ikezoe+T&cauthor_id=20382200), [Chie Nishioka](https://pubmed.ncbi.nlm.nih.gov/?term=Nishioka+C&cauthor_id=20382200) .Inhibition of mTORC1 by RAD001 (everolimus) potentiates the effects of 1,25-dihydroxyvitamin D(3) to induce growth arrest and differentiation of AML cells in vitro and in vivo. Exp Hematol. 2010 Aug;38(8):666-76. PMID: 20382200

**Reviewer 2: This is an interesting piece of work. The findings provide more insight on the role of 1.25(OH)2D3 on glomerulonephritis. The authors should check that the article is clear and concise. Grammatical errors were present in the abstract and manuscript.**

**Response:**

Thank you for your recognition and encouragement. We deeply evaluate and revise the language, grammar, and logic in the manuscript and invite professional scientific editing service (MedSci, Shanghai Metz Pharmaceutical Technology Co., Ltd) to help us，which meets PLOS One's requirements.

Thank you for your consideration and further review of our manuscript. Please do not hesitate to contact us with any further questions or recommendations.

Yours Sincerely,

Xiaoping Yang

Jianfeng Li

Lei Jin
