# Supplementary material for: 1.25(OH)2D3 decreases PCNA and mTOR expression and alleviates renal injury in Thy-1 nephritis rat model
Source: PLoS One. 2024 Dec 5;19(12):e0311000. doi: 10.1371/journal.pone.0311000 (PMC11620346; doi:10.1371/journal.pone.0311000)
Supplement: Attachment — Submitted filename: Response for Journal requirements .docx [file pone.0311000.s003.docx]

Dear editors,

Thank you very much for your requests for our manuscripts. We read the Journal requirements carefully and did a lot of work to meet the magazine's requirements.

Next, we responded to your questions one by one, and our responses are marked in red.

Journal Requirements:

Please review your reference list to ensure that it is complete and correct. If you have cited papers that have been retracted, please include the rationale for doing so in the manuscript text, or remove these references and replace them with relevant current references. Any changes to the reference list should be mentioned in the rebuttal letter that accompanies your revised manuscript. If you need to cite a retracted article, indicate the article’s retracted status in the References list and also include a citation and full reference for the retraction notice.

**Response:** *As requested by the magazine, we rechecked the format of the references， we ensure that our reference list is complete and correct and did not cited retracted article.*

*We uploaded figure files to the Preflight Analysis and Conversion Engine (PACE) digital diagnostic tool,* [*https://pacev2.apexcovantage.com/*](https://pacev2.apexcovantage.com/)*. And ensure that figures meet PLOS requirements.*

I am keen to hear from you soon and thank you again for your help.

With best regards,

Yours sincerely,

(Corresponding author)

Xiaoping Yang

Department of nephrology, The first affiliated hospital of Shi he zi University, Xin jiang province, China

E-mail: xjshzdxyxp@163.com

Tel: +86-13579458071
